# Long-term results from the AGILE study of azacitidine plus ivosidenib vs placebo in newly diagnosed *IDH1*-mutated AML^∗^

**DOI:** 10.1182/bloodadvances.2025016399

**Published:** 2025-07-28

**Authors:** Pau Montesinos, Dylan M. Marchione, Christian Recher, Michael Heuser, Susana Vives, Ewa Zarzycka, Jianxiang Wang, Marta Riva, Rodrigo T. Calado, Andre C. Schuh, Su-Peng Yeh, Adriana E. Tron, Jianan Hui, Diego A. Gianolio, Sung Choe, Prapti Patel, Stéphane De Botton, Courtney D. DiNardo, Hartmut Döhner

**Affiliations:** 1 Department of Hematology, Hospital Universitari i Politècnic La Fe, València, Spain; 2 Servier BioInnovation, Boston, MA; 3 Department of Hematology, Université de Toulouse, Institut Universitaire du Cancer de Toulouse Oncopole, CHU de Toulouse, Toulouse, France; 4 Department of Hematology, Hemostasis, Oncology and Stem Cell Transplantation, Hannover Medical School, Hannover, Germany; 5 Department of Hematology, Hospital Universitario Germans Trias i Pujol-ICO Badalona, Josep Carreras Research Institute, Universitat Autònoma de Barcelona, Badalona, Spain; 6 Department of Hematology and Transplantology, Medical University of Gdansk, Gdansk, Poland; 7 Institute of Hematology and Blood Diseases Hospital, Peking Union Medical College, Tianjin, China; 8 Department of Hematology, Oncology and Molecular Medicine, ASST Grande Ospedale Metropolitano Niguarda, Milan, Italy; 9 Department of Medical Imaging, Hematology and Oncology, Ribeirão Preto Medical School, University of São Paulo, São Paulo, Brazil; 10 Department of Medical Oncology and Hematology, Princess Margaret Cancer Centre, Toronto, ON, Canada; 11 Division of Hematology and Oncology, Department of Internal Medicine, China Medical University, Taichung, Taiwan; 12 Department of Hematology, Institut Gustave Roussy, Faculté Paris-Saclay, Villejuif, France; 13 Division of Cancer Medicine, Department of Leukemia, The University of Texas MD Anderson Cancer Center, Houston, TX; 14 Department of Internal Medicine III, Ulm University Hospital, Ulm, Germany

## Abstract

In the phase 3 AGILE study, after a 12.4-month median follow-up, ivosidenib, a mutant isocitrate dehydrogenase 1 (*IDH1*) inhibitor, combined with azacitidine significantly improved event-free survival, overall survival (OS), and complete remission rates compared with placebo-azacitidine in patients with newly diagnosed *IDH1*-mutated acute myeloid leukemia (AML), who were unfit for intensive chemotherapy. This post hoc analysis reports long-term follow-up results from AGILE after a median follow-up of 28.6 months. Overall, 148 patients were randomized to receive ivosidenib-azacitidine (n = 73) or placebo-azacitidine (n = 75). Median OS was significantly longer with ivosidenib (29.3 months; 95% confidence interval [CI], 13.2 to not reached) than with placebo (7.9 months; 95% CI, 4.1-11.3; hazard ratio, 0.42 [95% CI, 0.27-0.65]; *P* < .0001). Hematologic recovery was faster, more durable, and conversion to transfusion independence (53.8% vs 17.1%; *P* = .0004) was more common with ivosidenib than with placebo. Of 33 ivosidenib-treated patients evaluable for molecular measurable residual disease (MRD), 10 converted to MRD negativity. Although OS did not differ significantly between MRD-negative and MRD-positive responders at the 0.1% variant allele frequency (VAF) threshold, MRD-negative patients had numerically longer survival. MRD status appeared more predictive of long-term OS when an exploratory 1% VAF threshold was applied. MRD response was not associated with *IDH1* variant, VAF, inferred clonality, or number of baseline comutations. The previously reported safety profile was maintained. These long-term efficacy and safety results confirm the benefit of ivosidenib-azacitidine in this challenging-to-treat population and support its use as a standard of care with the longest reported survival benefit for intensive chemotherapy–ineligible patients with *IDH1*-mutated AML. This trial was registered at www.ClinicalTrials.gov as #NCT03173248.

## Key Points

• In long-term follow-up, ivosidenib-azacitidine, with a median OS of 29.3 months, sustained survival and hematologic benefits in mutant *IDH1* AML.
• Among patients with mutant *IDH1* AML in complete remission with ivosidenib-azacitidine, 30% experienced deep molecular clearance on treatment.

## Visual Abstract

**Figure:**
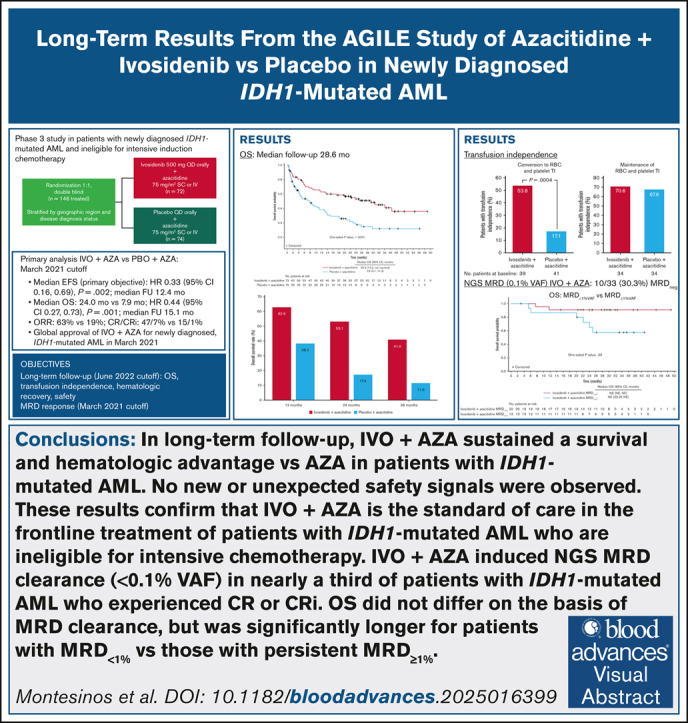

## Introduction

Acute myeloid leukemia (AML) is the most common form of acute leukemia to occur in adults, mainly affecting older individuals, with a median age of onset of 68 years.^1^, ^2^, ^3^ Treatment for older patients and patients unable to receive intensive induction chemotherapy (IC) is often limited to less-intensive, noncurative regimens due to comorbidities, compromised organ function, and poor performance status.^4^^,^^5^ Addition of the B-cell lymphoma 2 inhibitor venetoclax to low-intensity regimens improved overall survival (OS) in patients with mutation-agnostic AML who were ineligible for IC, but was associated with increased toxicity.^6^, ^7^, ^8^ Long-term survival in this population remains poor, and safer, more effective treatment strategies are needed.

Somatic mutations in the gene encoding isocitrate dehydrogenase 1 (*IDH1*) occur in 6% to 10% of patients with AML.^9^, ^10^, ^11^, ^12^ Mutant IDH proteins acquire a neomorphic enzymatic activity that allows the conversion of α-ketoglutarate to 2-hydroxyglutarate,^13^ which can lead to genomewide histone and DNA methylation alterations, impaired differentiation of myeloid lineage cells, and, ultimately, tumorigenesis.^14^^,^^15^ The most common *IDH1* mutation involves the critical amino acid residue R132 in the catalytic domain, with 5 variants identified in AML.^16^ *IDH1* mutation is an early event in AML that is associated with older age and poor prognosis.^7^^,^^11^^,^^17^^,^^18^

Ivosidenib is a potent, first-in-class oral-targeted inhibitor of mutant *IDH1* (m*IDH1*) that was approved in combination with azacitidine in 2022 in the United States for adults with newly diagnosed *IDH1*-mutated AML, who are aged ≥75 years or who have coexisting conditions that preclude intensive chemotherapy,^19^ and in 2023 in Europe for adults with newly diagnosed *IDH1*-mutated AML, who are not eligible to receive standard IC.^20^ Approval was based on the phase 3 AGILE study that compared ivosidenib-azacitidine with placebo-azacitidine in the frontline setting in patients with newly diagnosed *IDH1*-mutated AML who are not eligible for IC.^21^ The primary end point was event-free survival (EFS), defined as the time from randomization until treatment failure (lack of complete remission [CR] by week 24), relapse from remission, or death from any cause, whichever occurred first. In the primary analysis, after a median follow-up of 12.4 months, EFS was significantly longer in the ivosidenib-azacitidine arm than in the placebo-azacitidine arm (hazard ratio [HR], 0.33; 95% confidence interval [CI], 0.16-0.69; *P* = .002). After a median follow-up of 15.1 months, median OS was 24.0 months (95% CI, 11.3-34.1) and 7.9 months (95% CI, 4.1-11.3) in ivosidenib-azacitidine and placebo-azacitidine arms, respectively. Ivosidenib-treated patients had a higher CR rate compared with placebo-treated patients (47% vs 15%) and an increased rate of conversion to transfusion independence (46% vs 18%). Clinical responses to ivosidenib-azacitidine were associated with deep clearance of m*IDH1* and clearance of baseline mutations below the threshold of conventional next-generation sequencing (NGS; limit of detection [LOD], 2.0% variant allele frequency [VAF]).^22^^,^^23^ Acquired resistance to ivosidenib-azacitidine was associated with expansion or emergence of high-risk mutations occurring independently of m*IDH1*.^23^ Rates of febrile neutropenia and infections in the AGILE study were lower in the ivosidenib-azacitidine arm, whereas those of neutropenia and bleeding events were lower for placebo-azacitidine.

This study presents long-term OS and safety data from the AGILE study, and the depth of response to ivosidenib-azacitidine using NGS-based molecular measurable residual disease (MRD) analysis with a lower LOD (0.1%-0.5%) than the previous NGS analysis.

## Methods

### Patients and study design

Details of the global, double-blind, randomized, placebo-controlled, phase 3 AGILE study (ClinicalTrials.gov identifier: NCT03173248) have been published previously.^21^ Adults with untreated *IDH1*–mutated AML who were ineligible for intensive IC were randomized 1:1 to receive either 500 mg ivosidenib or placebo orally, once daily, each in combination with subcutaneous or IV 75 mg/m^2^ azacitidine for 7 days in 28-day cycles. Randomization was stratified by geographic region and disease status (primary vs secondary AML). Patients were treated for at least 6 cycles, unless relapse, disease progression, unacceptable toxicity, or death occurred. Patient enrollment started in March 2018 and was discontinued in May 2021 due to more deaths in the placebo-azacitidine arm than the ivosidenib-azacitidine arm. The data cutoff date for the primary trial analysis was March 2021.^21^ After study unblinding in July 2021, eligible patients in the placebo-azacitidine arm were allowed to cross over to ivosidenib-azacitidine treatment.

The trial protocol was approved by the study-site review boards or ethics committees and conducted according to the International Council for Harmonization Good Clinical Practice guidelines and the principles of the Declaration of Helsinki. All study patients gave written informed consent.

### Clinical end points and assessments

The primary end point was EFS and secondary end points included OS, rate of CR, CR and CR with partial hematologic recovery, CR and CR with incomplete hematologic recovery (CRi; included incomplete platelet recovery), duration of CR or CRi (DOR), transfusion requirements, and safety. EFS was defined as the time from randomization until treatment failure (lack of CR by week 24), relapse, or death, whichever occurred first. As in the original AGILE study, an exploratory “alternative EFS” analysis was conducted in which EFS was defined as the time from randomization until disease progression, relapse after CR or CRi, death, or treatment failure (lack of CR, CRi, or morphologic leukemia-free state by week 24). Transfusion independence was defined as ≥56 days without transfusion during study treatment plus 28 days, disease progression, confirmed relapse, death, or data cutoff, whichever occurred first.

After unblinding, bone marrow aspirates and biopsies beyond those performed per standard of care were no longer mandated per study protocol; as a result, the current post hoc analysis does not update EFS, CR rate, or DOR results. OS follow-up continued after study unblinding, and all patients alive after an EFS event were monitored every 8 weeks for survival. Peripheral blood samples for hematology and biochemistry were collected during screening, every week in cycle 1, every other week in cycles 2 to 4, and every month thereafter. Samples were analyzed at local sites according to International Committee for Standardization in Hematology guidelines.

Adverse events (AEs) were graded according to the National Cancer Institute Common Terminology Criteria for Adverse Events, version 4.03.

### MRD assessments

MRD assessment was performed at the Munich Leukemia Laboratory (GmbH) on DNA extracted from cryopreserved bone marrow mononuclear cells; suggested time points included day 1 of cycles 3, 5, 7, 9, 11, 14, 20, 26, and 32. Baseline DNA was analyzed using a diagnostic 51-gene myeloid NGS panel (LOD, 3% VAF; supplemental Table 1). A subsequent on-treatment NGS analysis of MRD was performed with a 26-gene error-corrected AML MRD panel (supplemental Table 2) using unique molecular identifiers, as recommended by the European LeukemiaNet (ELN) MRD Working Party Consensus.^24^

An MRD NGS Enrichment Library Prep Kit (Twist Bioscience, South San Francisco, CA) with a unique molecular identifier adapter system was used on a manufacturer-designed AML panel consisting of the following genes: *ASXL1*, *CALR*, *CEBPA*, *DDX41*, *DNMT3A*, *ETV6*, *EZH2*, *FLT3*, *IDH1*, *IDH2*, *JAK2*, *KIT*, *KRAS*, *MPL*, *NPM1*, *NRAS*, *PTPN11*, *RAD21*, *RUNX1*, *SF3B1*, *SRSF2*, *STAG2*, *TET2*, *TP53*, *U2AF1*, and *WT1*.

The methods for library processing are described in the supplemental Methods. At least 15 reads per read family to accept a given fragment and 400× coverage per read family were required to call a variant. For variants detected at baseline, the detection sensitivity in subsequent samples was 0.1% MRD VAF; for mutations not detected at baseline or if baseline samples were unavailable, the detection sensitivity was 0.5% MRD VAF. All variants above the applicable detection sensitivity threshold with known or potential clinical significance (tier I or tier II)^25^ were considered to be evidence of MRD, except for *DNMT3A*, *TET2*, and *ASXL1* (*DTA)* mutations, which were excluded to reduce the risk of false-positive MRD due to clonal hematopoiesis. The *IDH1* mutation was considered dominant if the *IDH1* VAF was higher than that of all other non-*DTA* variants in the individual patient.

A patient was classified as MRD-negative (MRD_neg_) and reported to demonstrate deep molecular clearance if a reduction of all baseline non*-DTA* mutations <0.1% MRD VAF and no new emerging mutations >0.5% MRD VAF were observed. If either criterion was not met, patients were considered to have an MRD-positive (MRD_pos_) response. Exploratory analyses of DOR, EFS per protocol, alternative EFS, and OS were performed using a 1% MRD VAF threshold for both reduction of baseline and emerging mutations.

### Analysis populations

The data cutoff for the updated OS, hematology, and transfusion dependence analyses was June 2022. OS and transfusion independence were analyzed in the intention-to-treat (ITT) population, which included all randomized patients. All patients who received ≥1 dose of study treatment were included in safety and hematologic assessments.

MRD-evaluable patients included those in the primary AGILE analysis who had the best overall response of CR or CRi and ≥1 on-treatment bone marrow mononuclear cell sample with a corresponding response assessment. MRD responses and EFS data for subanalyses were based on the primary data cutoff on March 2021; OS and patient disposition data were based on the June 2022 data cutoff.

### Statistical analysis

OS was estimated using the Kaplan-Meier method, with point estimates and 95% CI; treatment arms were compared using the log-rank test stratified by geographic region and disease status. The HR of OS was estimated using a Cox proportional hazards model with geographic region and disease status as stratification factors. For Kaplan-Meier OS estimates by baseline *IDH1* VAF, the “high” subgroup had baseline *IDH1* VAF greater than or equal to median for that treatment arm, whereas “low” had baseline *IDH1* VAF less than median. Transfusion independence was compared between arms using the Cochran-Mantel-Haenszel test, with the same stratification factors. No statistical adjustments were made for treatment crossover. A 2-sided Wilcoxon test was used to determine differences in MRD response according to baseline m*IDH1* VAF and number of variants. A 1-sided log-rank test, stratified by randomization factors of AML status and geographic region, was used to evaluate OS, EFS, and DOR at 0.1% and 1.0% MRD VAF thresholds. Fisher exact test was used to determine correlations between inferred m*IDH1* clonality or specific gene alterations and MRD response.

## Results

### ITT population

**Figure 1.**
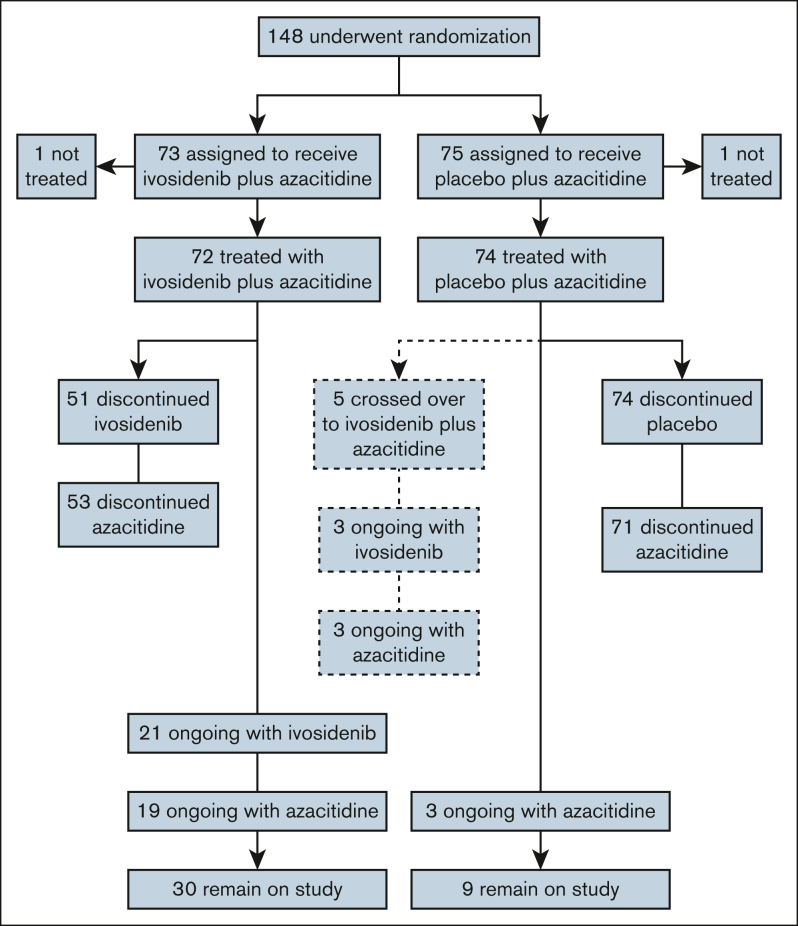
**Patient disposition in the ITT population.**

The ITT population comprised 148 patients, 2 of whom had been randomized after the primary analysis. Of these, 73 patients were randomized to ivosidenib-azacitidine and 75 to placebo-azacitidine; 1 patient in each arm did not receive treatment (Figure 1). Thirty-five (49%) patients treated with ivosidenib-azacitidine, and 42 (58%) treated with placebo-azacitidine received concomitant azole for a median of 1 month. No dose adjustments were made to ivosidenib because of azole use. Baseline characteristics were well balanced between the treatment arms (supplemental Table 3; supplemental Figure 1), and consistent with those previously reported for 146 patients in the primary publication. Five patients crossed over from placebo to ivosidenib treatment after unblinding. At the June 2022 data cutoff, most patients before crossover had discontinued treatment with ivosidenib or placebo, most frequently due to AEs (29%), progression or relapse (22%), or patient withdrawal (11%). Among the 5 crossover patients, 3 continued on treatment, 1 withdrew from the study, and 1 died after 300 days of treatment with ivosidenib. The median treatment duration was 10.8 months for the ivosidenib-azacitidine arm and 3.2 months for the placebo-azacitidine arm. For the 5 crossover patients, the median ivosidenib treatment duration following crossover was 8.6 months. After discontinuation of AGILE study treatment, 19 patients (26.0%) in the ivosidenib-azacitidine arm and 23 (30.7%) in the placebo-azacitidine arm received at least 1 subsequent anticancer therapy (supplemental Table 4).

### MRD-evaluable population

The MRD-evaluable population comprised 33 patients in the ivosidenib-azacitidine arm and 10 in the placebo-azacitidine arm; the analysis set comprised 173 samples (supplemental Figure 2). All patients had ≥1 baseline mutation in the 26-gene MRD panel. The median number of baseline mutations was 4 (range, 1-10) per patient and the median number of MRD assessments was 3 (range, 1-9); the median follow-up period was 189 days (range, 49-875). No patients had baseline mutations in *IDH2.*

### OS, hematology, and transfusion dependence

**Figure 2.**
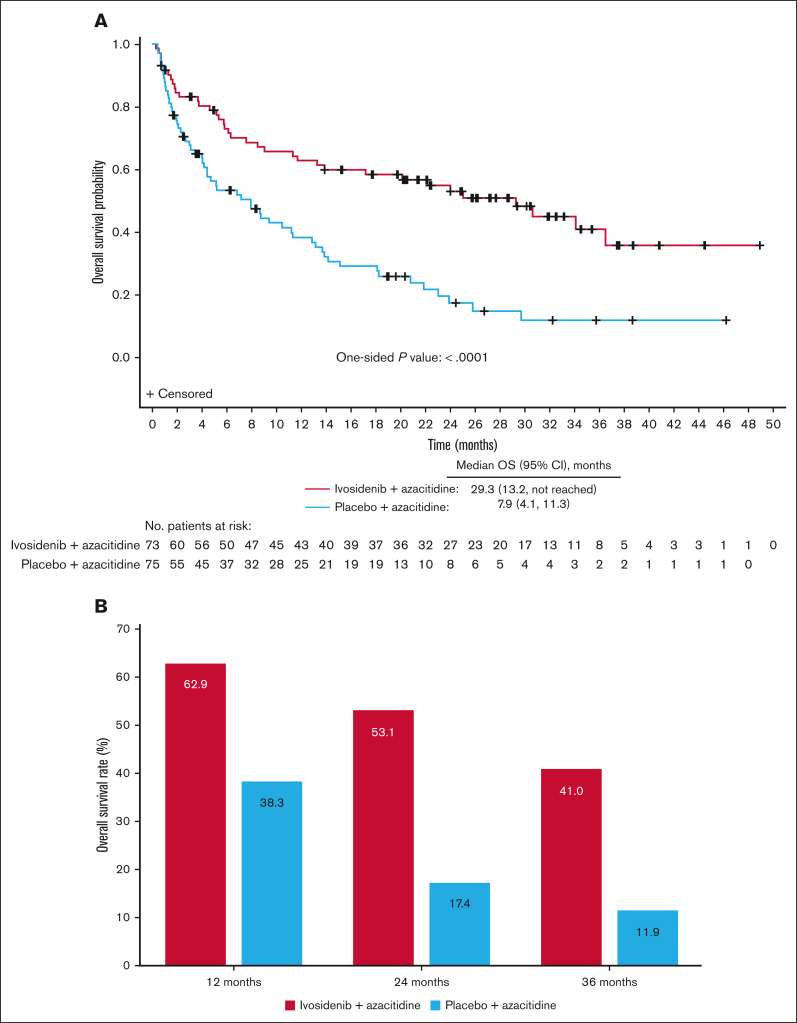
**OS in patients treated with ivosidenib + azacitidine or placebo + azacitidine in the ITT population.** (A) Kaplan-Meier estimates of OS with a median follow-up of 28.6 months. (B) OS rates at 12, 24, and 36 months.

At a median follow-up of 28.6 months, median OS was 29.3 months (95% CI, 13.2 to not reached) for ivosidenib-treated patients and 7.9 months (95% CI, 4.1-11.3) for placebo-treated patients (Figure 2A). The difference between treatment arms was significant, with an HR of 0.42 (95% CI, 0.27-0.65) and 1-sided *P* value of <.0001. There were 95 OS events, 37 in the ivosidenib-azacitidine arm and 58 in the placebo-azacitidine arm, and OS rates over time were consistently higher in the ivosidenib arm (Figure 2B). OS was particularly poor for patients with high baseline *IDH1* VAF treated with placebo-azacitidine; however, OS was notably improved in ivosidenib-treated patients irrespective of baseline *IDH1* VAF (supplemental Figure 3).

**Figure 3.**
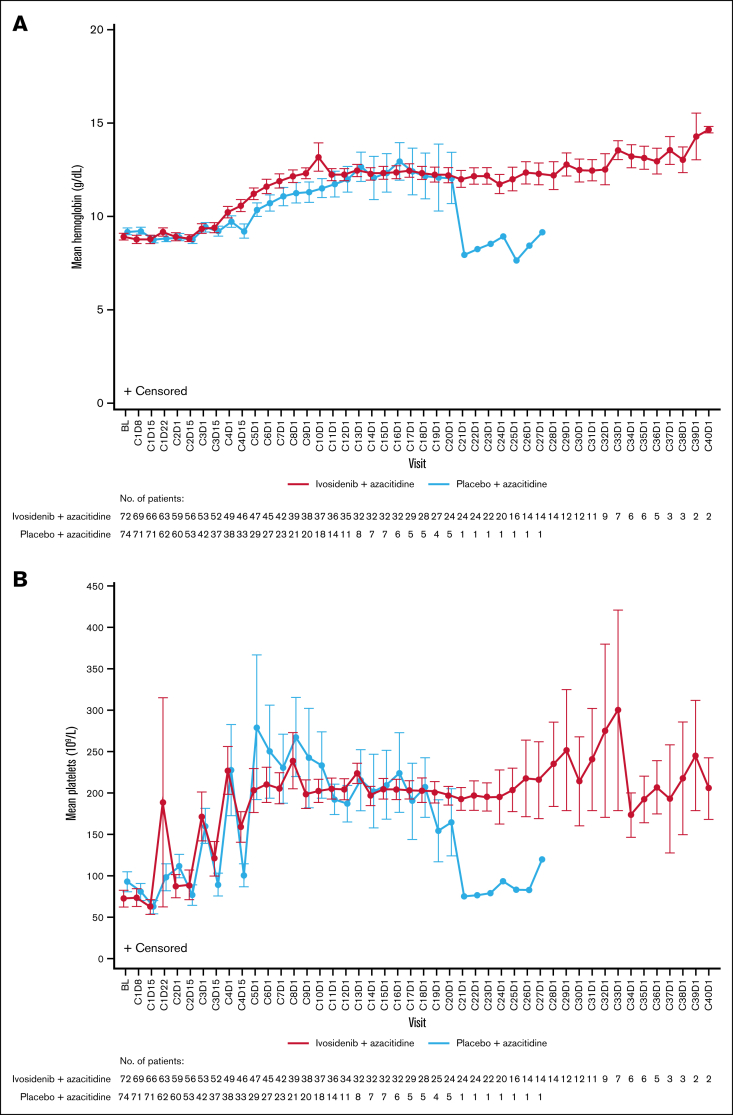

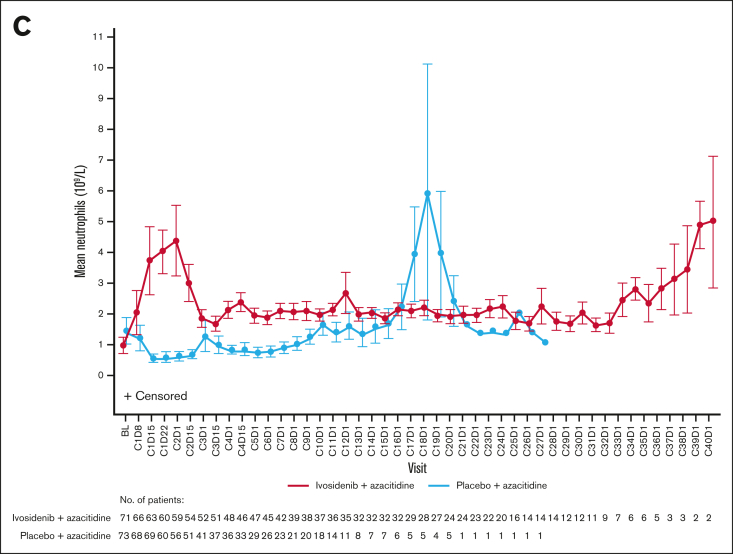
**Hematology outcomes for patients treated with ivosidenib + azacitidine or placebo + azacitidine in the ITT population.** Mean ± standard error hemoglobin (A), platelet counts (B), and neutrophil counts (C) over time. BL, baseline; C1D8, cycle 1 day 8.

Hematologic recovery occurred generally faster and lasted longer with ivosidenib-azacitidine compared with placebo-azacitidine (Figure 3). In the ivosidenib-azacitidine arm, median hemoglobin levels increased steadily from baseline and stabilized at ∼12 g/dL from around cycle 6 (supplemental Figure 4A). Median platelet counts recovered from baseline values to ∼180 × 10^9^/L and then remained stable from cycle 4 onward (supplemental Figure 4B). Median neutrophil counts rapidly increased from baseline to cycle 1 day 22 and stabilized within the normal range from cycle 4 onward (supplemental Figure 4C). In the placebo-azacitidine arm, platelet count recovery was similar to the ivosidenib-azacitidine arm, whereas neutrophil count and hemoglobin level recovery was less pronounced and delayed, with normal levels not reached until cycles 13 and 16, respectively. More patients in the ivosidenib-azacitidine arm had hematologic recovery, pointing to the greater efficacy of ivosidenib-azacitidine vs placebo-azacitidine.

**Figure 4.**
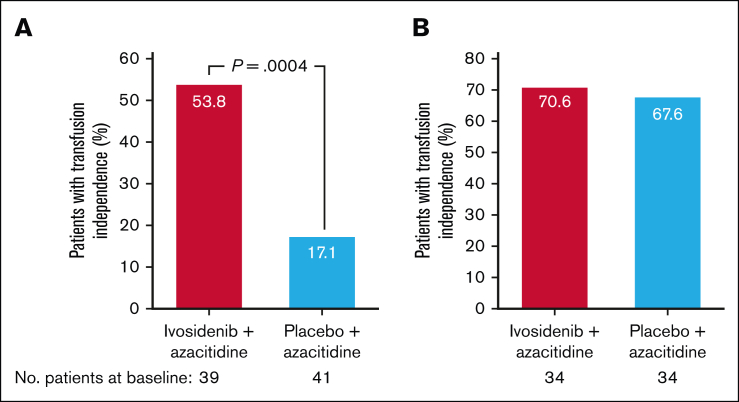
**Red blood cell and platelet transfusion independence in the ITT population.** (A) Rate of conversion to red blood cell and platelet transfusion independence. (B) Maintenance of red blood cell and platelet transfusion independence from baseline.

At baseline, 53.4% of patients were red blood cell and/or platelet transfusion dependent in the ivosidenib-azacitidine arm and 54.7% in the placebo-azacitidine arm. A greater proportion of these patients became both red blood cell and platelet transfusion independent during treatment with ivosidenib-azacitidine (21/39 [53.8%]) compared with placebo-azacitidine (7/41 [17.1%]; 1-sided *P* = .0004; Figure 4A). Among patients who were transfusion independent at baseline, the proportion who maintained this was similar between the ivosidenib-azacitidine and placebo-azacitidine arms (24/34 [70.6%] vs 23/34 [67.6%]; Figure 4B).

### MRD outcomes in ivosidenib-azacitidine–treated patients

Of 33 MRD-evaluable patients in the ivosidenib-azacitidine arm, 10 (30.3%) converted to an MRD_neg_ response by day 1 of cycle 14, all of whom had CR (supplemental Figure 5). Of these patients, 7 (70%) had converted to an MRD_neg_ response by day 1 of cycle 7. Of the 23 patients who remained MRD_pos_, 19 had CR and 4 CRi. Two (20.0%) patients had an MRD_neg_ response in the placebo-azacitidine arm (supplemental Figure 2). This group was excluded from further analysis due to small patient numbers.

There were no significant differences in baseline characteristics between MRD_pos_ and MRD_neg_ patients (supplemental Table 5). Moreover, no significant differences in MRD response rates were observed according to baseline m*IDH1* VAF, inference of m*IDH1* clonality, or the baseline number of distinct variants (supplemental Figure 6). Refer to supplemental Results for non-MRD panel baseline mutations in 4 MRD_neg_ patients. Most baseline variants decreased over time, with no single gene showing mutations associated with statistically significant differences in MRD response (supplemental Table 6; supplemental Figure 7). All 5 (15.2%) of the 33 patients with confirmed clinical relapses had detectable MRD at their last completed assessment prior to relapse (supplemental Figure 8).

**Figure 5.**
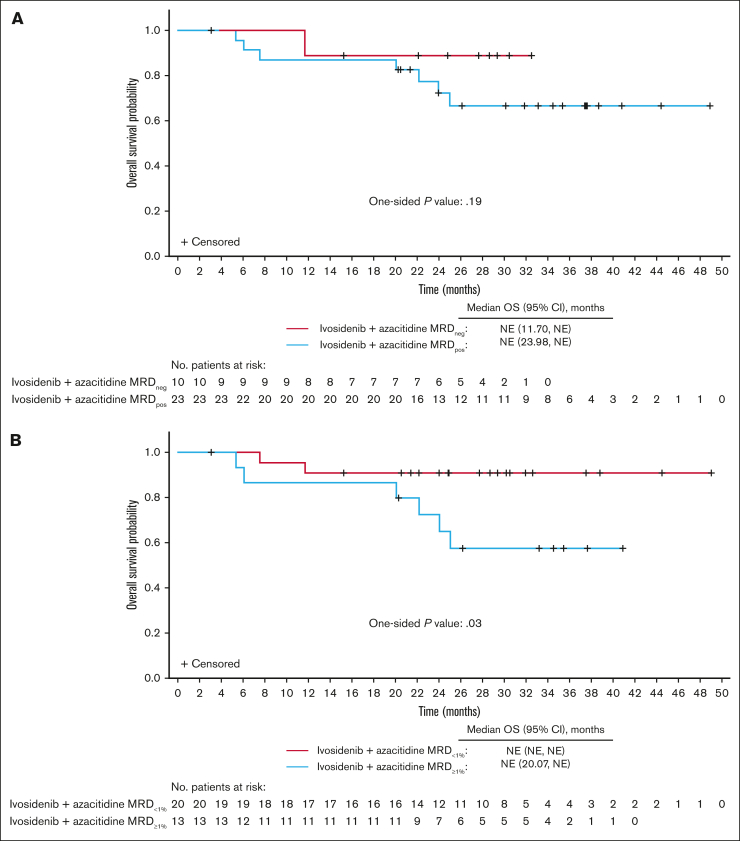
**OS in MRD-evaluable patients treated with ivosidenib + azacitidine according to MRD status (n = 33).** (A) OS in MRD_neg_ vs MRD_pos_ patients. (B) OS in patients with MRD_<1%_ vs patients with MRD_≥1%_. NE, not evaluable.

Although no significant differences were observed in estimates of OS (1-sided *P* = .19; Figure 5A), DOR, EFS, or alternative EFS between MRD_neg_ and MRD_pos_ responders, there was a trend toward longer outcomes in the MRD_neg_ group (supplemental Figures 9A,C and 10A). Although neither group reached the median OS, the OS range for censored patients was 3.1 to 32.5 months among MRD_neg_ responders and 5.4 to 48.9 months among MRD_pos_ responders.

In additional analyses, patients were stratified on the basis of reduction of baseline mutations to <1% MRD VAF (MRD_<1%_; MRD_<1%_ vs MRD_≥1%_ [baseline mutations ≥1% MRD VAF] responders). Furthermore, the threshold for emerging mutations was adjusted to ≥1% MRD VAF. Of the 33 MRD-evaluable patients, as many as 20 (60.6%) were MRD_<1%_ responders, all having a best overall response of CR. Baseline characteristics were generally similar between MRD_<1%_ and MRD_≥1%_ responders, except more MRD_<1%_ responders had de novo AML (85.0% vs 53.8%; supplemental Table 7). Notably, the OS estimate was significantly longer in MRD_<1%_ than in MRD_≥1%_ responders (1-sided *P* = .03; Figure 5B), as were the estimates of DOR, EFS, and alternative EFS (supplemental Figures 9B,D and 10B).

### Safety

Among the most common hematologic AEs, anemia occurrence was similar in both treatment arms (ivosidenib-azacitidine, 33.3%; placebo-azacitidine, 31.1%), whereas neutropenia (30.6% vs 21.6%) and thrombocytopenia (29.2% vs 18.9%) were more common with ivosidenib-azacitidine, and febrile neutropenia (27.8% vs 33.8%) was more frequent with placebo-azacitidine (Table 1). The most common nonhematologic AEs in all patients were constipation (ivosidenib-azacitidine, 31.9%; placebo-azacitidine, 52.7%), nausea (44.4% vs 39.2%), and pyrexia (37.5% vs 43.2%). The incidence of total infection events, and pneumonia specifically, was higher in the placebo-azacitidine arm (51.4%, 32.4%) compared with the ivosidenib-azacitidine arm (34.7%, 23.6%). All-grade bleeding events had a higher incidence in the ivosidenib-azacitidine arm (43.1%) compared with the placebo-azacitidine arm (31.1%), but grade ≥3 bleeding was similar in both arms (6.9% vs 8.1%). AEs of special interest occurred more frequently in the ivosidenib-azacitidine arm; QT prolongation, differentiation syndrome, and leukocytosis were reported in 29.2%, 13.9%, and 12.5% in this arm, respectively, compared with 12.2%, 8.1%, and 2.7% in the placebo-azacitidine arm (Table 1). AEs leading to treatment discontinuation occurred in 19 (26.4%) ivosidenib-treated patients and 19 (25.7%) placebo-treated patients. Ivosidenib or placebo dose reductions due to AEs were necessary in 13 (18.1%) and 6 (8.1%) patients, respectively. Ivosidenib was interrupted in 23 patients (31.9%) and placebo in 29 patients (39.2%). In total, 34 patients experienced AEs that led to death, 11 (15.3%) in the ivosidenib-azacitidine arm and 23 (31.1%) in the placebo-azacitidine arm; none were considered related to treatment.

**Table 1.** **AEs in the safety population**

Event, n (%)	Ivosidenib + azacitidine n = 72		Placebo + azacitidine n = 74	
	Any grade	Grade ≥3	Any grade	Grade ≥3
**Hematologic AEs (in ≥10% of patients in either arm)**				
Anemia	24 (33.3)	19 (26.4)	23 (31.1)	20 (27.0)
Neutropenia	22 (30.6)	22 (30.6)	16 (21.6)	16 (21.6)
Thrombocytopenia	21 (29.2)	18 (25.0)	14 (18.9)	14 (18.9)
Febrile neutropenia	20 (27.8)	20 (27.8)	25 (33.8)	25 (33.8)
Decreased platelet count	10 (13.9)	8 (11.1)	6 (8.1)	6 (8.1)
Decreased neutrophil count	9 (12.5)	9 (12.5)	5 (6.8)	5 (6.8)
Leukocytosis	8 (11.1)	0	2 (2.7)	1 (1.4)
**Nonhematologic AEs (in ≥20% of patients in either arm)**				
Nausea	32 (44.4)	2 (2.8)	29 (39.2)	3 (4.1)
Vomiting	30 (41.7)	0	20 (27.0)	1 (1.4)
Pyrexia	27 (37.5)	2 (2.8)	32 (43.2)	2 (2.7)
Diarrhea	26 (36.1)	1 (1.4)	29 (39.2)	6 (8.1)
Constipation	23 (31.9)	0	39 (52.7)	1 (1.4)
Pneumonia	17 (23.6)	16 (22.2)	24 (32.4)	22 (29.7)
Electrocardiogram QT prolonged	16 (22.2)	8 (11.1)	5 (6.8)	2 (2.7)
Decreased appetite	13 (18.1)	1 (1.4)	21 (28.4)	6 (8.1)
Asthenia	12 (16.7)	0	25 (33.8)	6 (8.1)
Hypokalemia	11 (15.3)	2 (2.8)	21 (28.4)	7 (9.5)
Peripheral edema	9 (12.5)	0	17 (23.0)	1 (1.4)
Any infection	25 (34.7)	16 (22.2)	38 (51.4)	23 (31.1)
Any bleeding	31 (43.1)	5 (6.9)	23 (31.1)	6 (8.1)
**AEs of special interest**				
QT prolongation	21 (29.2)	9 (12.5)	9 (12.2)	3 (4.1)
Differentiation syndrome	10 (13.9)	3 (4.2)	6 (8.1)	3 (4.1)
Leukocytosis	9 (12.5)	0	2 (2.7)	1 (1.4)

## Discussion

This follow-up of the AGILE trial data demonstrates the long-term clinical benefit of ivosidenib-azacitidine compared with placebo-azacitidine in patients with newly diagnosed *IDH1*-mutated AML who were unfit for IC, including sustained OS advantage and hematologic recovery. After a median follow-up of 28.6 months, the median OS was significantly longer in ivosidenib-treated patients than placebo-treated patients (29.3 vs 7.9 months; HR, 0.42; 1-sided *P* < .0001). Of note, the median OS is now >5 months longer than that reported in the primary analysis of ivosidenib-azacitidine treatment (24 months), with a greater risk-reduction of death compared with the placebo-azacitidine arm. Conversion to transfusion independence from baseline has also increased with longer follow-up, from 46% to 54% in the ivosidenib-azacitidine arm.^21^

Ivosidenib-azacitidine induced NGS-based molecular MRD negativity in approximately one-third of MRD-evaluable patients, the majority of whom converted to an MRD_neg_ response by cycle 7 day 1. MRD response rates assessed by multiparameter flow cytometry have been similar in other studies of hypomethylating agent–based doublet treatment (venetoclax-azacitidine or venetoclax-decitabine), and higher for the ivosidenib-azacitidine-venetoclax triplet (83.3%).^26^, ^27^, ^28^, ^29^ In our analysis, MRD_neg_ responses were observed across a range of patient and disease characteristics and occurred irrespective of diagnostic m*IDH1* VAF, inferred clonality of m*IDH1* based on relative VAFs at diagnosis, or the number of baseline variants, although this lack of difference should be interpreted with caution due to low patient numbers. Other limitations of the molecular analyses included the lack of uniformity among time points for MRD testing across patients, and the limited number of variants that can be tracked with sufficient sensitivity in any panel-based NGS MRD assessment. The latter meant that the MRD gene panel in the study could not include all variants known from diagnosis.

Molecular response using the 0.1% threshold was not associated with differences in OS, in contrast to other studies of less-intensive treatments that used multiparameter flow cytometry–based methods.^26^^,^^29^ This could be related to the limited number of MRD-evaluable patients and samples, or to the fact that some patients could have demonstrated MRD_neg_ responses between the March 2021 and June 2022 cutoffs (sample collection was discontinued after study unblinding in March 2021). When the MRD VAF threshold was relaxed to 1%, the probability of OS significantly favored patients with MRD_<1%_ vs those with persistent MRD_≥1%_, and a substantial proportion of patients with MRD_<1%_ experienced prolonged remission and survival. Although the provisional NGS MRD threshold of 0.1% VAF has proven useful in studies of younger patients treated with IC,^1^^,^^24^^,^^30^^,^^31^ a 1% VAF threshold may have better prognostic value in older patients with AML undergoing less-intensive treatment. These observations should be explored in larger studies, especially given that *P* values were not adjusted for multiplicity and the sample size was small.

The long-term safety profile of ivosidenib-azacitidine was consistent with that previously reported and there were no new or unexpected safety signals or additional treatment discontinuations due to AEs compared with the primary analysis.^21^ The rates of differentiation syndrome of any grade with ivosidenib-azacitidine remained stable, indicating early occurrence and potentially minimal impact on long-term treatment with ivosidenib. The rate of any-grade (29%) or grade ≥3 (13%) QT interval prolongation increased somewhat compared with the earlier analysis (any grade, 20%; grade ≥3, 10%), potentially indicating a cumulative effect with drug exposure, similar to the cumulative increase of cardiotoxic events observed in patients with AML in routine practice.^32^ The incidence of febrile neutropenia and infections remained lower with ivosidenib-azacitidine, whereas neutropenia and bleeding events remained less common with placebo-azacitidine. The higher neutropenia rate may be explained by the longer median duration of ivosidenib treatment compared with placebo (10.8 months vs 3.2 months), which resulted in discontinuation or dose reduction of long-term azacitidine in many patients. The lower rate of febrile neutropenia and infections among patients receiving ivosidenib-azacitidine might be due to the lower proportion of patients in this group who experienced relapse or developed refractory AML, as well as to accelerated initial neutrophil recovery (probably related to differentiation under ivosidenib).

Employing validated targeted agents for *IDH*-specific mutations is crucial to maximize patient outcomes. AGILE is the only phase 3 study to date specifically designed to assess a targeted therapy in the frontline setting in patients with *IDH1*-mutated AML. In subanalyses of clinical data for venetoclax in the phase 3 VIALE-A trial, survival benefit and remission rates with venetoclax-azacitidine treatment were greater for patients with *IDH2*-mutated AML (median 27.5 vs 13 months with placebo-azacitidine) than *IDH1*-mutated AML (median 10.2 vs 2.2 months with placebo-azacitidine).^7^^,^^33^ These results suggest that these mutations may be biologically distinct and should be assessed separately in clinical studies, highlighting the need for early systematic screening of *IDH1* mutations in the frontline setting for unfit patients with AML. Comparison of the long-term outcomes from the AGILE and VIALE-A studies, taken together with real-world evidence from a retrospective chart review of nearly 300 patients treated with venetoclax-azacitidine in the United States,^8^ support the 2022 ELN recommendation of ivosidenib-azacitidine treatment for patients with *IDH1*-mutated AML who are not eligible to receive IC.^24^ Toxicity data may also favor ivosidenib-azacitidine, with a decreased incidence of cytopenias, febrile neutropenia, and infections, although rates of QT prolongation and differentiation syndrome were higher with ivosidenib-azacitidine.^7^

Importantly, the new 2024 ELN genetic classification for prognostic stratification of patients receiving hypomethylating agents categorizes patients with *IDH1*-mutated AML who are treated with ivosidenib-azacitidine as having a favorable risk profile even in the presence of comutations in *DNMT3A*, *SRSF2*, *RUNX1*, or receptor tyrosine kinase genes.^34^ This is supported by the current finding that no single gene with baseline mutations was associated with a significant difference in MRD response. This contrasts with venetoclax-based regimens, where gene mutations in the receptor tyrosine kinase pathway, including *FLT3* internal tandem duplications, *KRAS,* and *NRAS,* are associated with inferior outcomes.^34^^,^^35^

Clinical trials are currently ongoing to further investigate ivosidenib treatment in patients unfit for IC^28^^,^^36^ as well as those eligible for IC.^37^

In conclusion, the long-term follow-up data from the AGILE study demonstrate a clear and robust clinical benefit of ivosidenib-azacitidine treatment in the challenging-to-treat population of patients with newly diagnosed *IDH1*-mutated AML who are unfit for IC. Deep molecular clearance was observed in approximately one-third of patients in CR, independent of *IDH1* variant, VAF, clonality, or number of comutations. This targeted therapy is an effective and safe option resulting in sustained survival in a patient population with still-poor prognosis when treated by conventional therapy, highlighting the need to wait for *IDH1* testing results to inform optimal treatment decision.

Conflict-of-interest disclosure: P.M. reports consulting or advisory roles with Servier, Bristol Myers Squibb (BMS), and Novartis; participation in speakers’ bureaus for Servier, BMS, Jazz Pharmaceuticals, Sanofi, AbbVie, and Teva Pharmaceuticals; research funding from BMS, 10.13039/100006483AbbVie, and Daiichi Sankyo; travel, accommodations, or expenses from 10.13039/100004319Pfizer. C.R. reports consulting or advisory roles with AbbVie, Amgen, Astellas, BMS, Boehringer, Jazz Pharmaceuticals, Johnson & Johnson, and Servier; research funding from 10.13039/100006483AbbVie, 10.13039/100002429Amgen, 10.13039/100004324Astellas, BMS, IQVIA, and Jazz Pharmaceuticals; and support for attending meetings and/or travel from 10.13039/100006483AbbVie, 10.13039/100004336Novartis, and 10.13039/501100011725Servier. M.H. reports research funding to institution from 10.13039/100006483AbbVie, 10.13039/100004326Bayer Pharma AG, 10.13039/100011096Jazz Pharmaceuticals, Glycostem, Karyopharm, PinotBio, 10.13039/501100011725Servier, and Toray; honoraria from 10.13039/100004324Astellas, 10.13039/501100022274Daiichi Sankyo, Janssen, Miltenyi, 10.13039/100019120Otsuka, Qiagen, and Servier; and consulting roles with 10.13039/100006483AbbVie, AvenCell, Ascentage Pharma, BMS, Janssen, 10.13039/100011096Jazz Pharmaceuticals, LabDelbert, 10.13039/100004336Novartis, 10.13039/100004319Pfizer, and 10.13039/501100011725Servier. S.V. reports travel, accommodations, and expenses from Astellas Pharma, Pfizer, Servier, AbbVie, and Jazz Pharmaceuticals; research funding from Astellas Pharma; consulting or advisory roles (without honoraria) with 10.13039/501100004948Astellas Pharma, 10.13039/100004319Pfizer, 10.13039/501100011725Servier, 10.13039/100006483AbbVie, and Jazz Pharmaceuticals. J.W. reports consulting or advisory role with AbbVie. R.T.C. reports honoraria from Novartis and Alexion Pharmaceuticals. A.C.S. reports clinical trials/research support: AbbVie, Amgen, Astellas, BMS, GlycoMimetics, J&J, Kite/10.13039/100005564Gilead, Loxo, 10.13039/100004336Novartis, 10.13039/100004319Pfizer, 10.13039/501100011725Servier, and Syndax; and participation on advisory board for 10.13039/100006483AbbVie, 10.13039/100002429Amgen, 10.13039/100004324Astellas, BMS, Jazz, Kite/10.13039/100005564Gilead, 10.13039/100004336Novartis, Paladin, Phebra, 10.13039/100004319Pfizer, 10.13039/501100011725Servier, and Teva. S.P.Y. reports honoraria from AbbVie, Amgen, Astellas Pharma, Bayer, BMS, Janssen, Novartis, Sanofi, and Takeda; and consulting or advisory roles with AbbVie, Chugai Pharma, Janssen, Novartis, Pfizer, and Sanofi. D.M.M., A.E.T., J.H., D.A.G., S.C., and P.P. are employees of Servier BioInnovation. S.D.B. reports honoraria from BMS, AbbVie, Servier, Jazz Pharmaceuticals, Astellas Pharma, and Loxo; consultancy or advisory roles with Servier, BMS, GlaxoSmithKline, Syndax, and Remix; participation in speakers’ bureaus for Servier, BMS, Jazz Pharmaceuticals, Astellas Pharma, and AbbVie; research funding from Forma and Auron; and travel, accommodations, or expenses from AbbVie and Servier. C.D.D. reports honoraria from AbbVie/Genentech, Astellas, BMS, Fogham, Notable Labs, ImmuneOnco, Servier, Novartis, and Takeda; and has received consultancy fees from Schrödinger. H.D. reports consulting or advisory roles with AbbVie, AstraZeneca, Gilead, Janssen, Jazz, Pfizer, Servier, Stemline Therapeutics, and Syndax; travel, accommodations, and expenses from AbbVie and Servier; and research funding from Amgen, BMS, Novartis, Pfizer, Jazz Pharmaceuticals, AbbVie, and Kronos Bio. The remaining authors declare no competing financial interests.

The current affiliation for M.H. is Department of Internal Medicine IV, University Hospital Halle (Saale), Martin-Luther-University Halle-Wittenberg, Halle, Germany.

## Supplementary Material

Supplemental Methods, References, Tables, and Figures
