# Supplementary material for: Long-term results from the AGILE study of azacitidine plus ivosidenib vs placebo in newly diagnosed IDH1-mutated AML
Source: Blood Adv. 2025 Jul 28;9(20):5177–89. doi: 10.1182/bloodadvances.2025016399 (PMC12550153; doi:10.1182/bloodadvances.2025016399)

Long-term results from the AGILE study of azacitidine plus ivosidenib vs placebo in newly diagnosed *IDH1*-mutated AML

Supplemental material

Supplemental methods for the MRD analyses 3

Supplemental results for the MRD analyses 6

Supplemental Table 1. 51-gene AML diagnostic panel (gene/region of interest) 7

Supplemental Table 2. Munich Leukemia Laboratory AML MRD 26-gene panel (gene/region of interest) 8

Supplemental Table 3. Baseline demographic and clinical characteristics in the ITT population 9

Supplemental Table 4. Subsequent anticancer therapies in the ITT population 11

Supplemental Table 5. Baseline demographics and disease characteristics in ivosidenib-azacitidine–treated patients in the MRD-evaluable population, overall and according to MRD response at the 0.1% threshold... 12

Supplemental Table 6. Presence or absence of baseline mutations and correlation with remaining MRD_{pos} or converting to MRD negativity 13

Supplemental Table 7. Baseline demographics and disease characteristics in ivosidenib-azacitidine–treated MRD-evaluable patients according to MRD response at a 1% VAF threshold 14

Supplemental Figure 1. Variant allele frequency distribution of *IDH1* mutations in bone marrow in the ITT population 15

Supplemental Figure 2. Disposition of patients in the MRD analyses 16

Supplemental Figure 3. Overall survival in patients treated with ivosidenib-azacitidine and placebo-azacitidine according to baseline *IDH1* VAF 17

Supplemental Figure 4. Hematology outcomes for patients treated with ivosidenib-azacitidine or placebo-azacitidine 18

Supplemental Figure 5. Time to MRD_{neg} response in ivosidenib-azacitidine–treated patients (N=10)............. 20

Supplemental Figure 6. Best MRD response in ivosidenib-azacitidine–treated MRD-evaluable patients (N=33) according to baseline mutation status 21

Supplemental Figure 7. Longitudinal evaluation of genes mutated at baseline and present in at least 1 patient in ivosidenib-azacitidine–treated MRD-evaluable patients (N=33).. 22

Supplemental Figure 8. Swimlane plot of MRD status over time in ivosidenib-azacitidine–treated MRD-evaluable patients (N=33). 23

Supplemental Figure 9. Duration of clinical outcomes in ivosidenib-azacitidine–treated MRD-evaluable patients according to MRD status (N=33).. 24

Supplemental Figure 10. Exploratory analysis for an alternative definition of EFS according to MRD response in ivosidenib-azacitidine–treated patients (N=33). 26

Supplemental methods for the MRD analyses

Details on the error-corrected NGS technique

Standard next-generation sequencing (NGS) is associated with intrinsic sequencing error rates, limiting variant calling at a 3–5% variant allele frequency (VAF). This VAF threshold is acceptable for diagnostic purposes but is inadequate for measurable residual disease (MRD) detection.[1] Error-corrected NGS utilizes physical incorporation of unique molecular identifiers (UMIs) at the library preparation step, prior to DNA amplification.[1,2] This early incorporation of UMIs minimizes the effect of errors generated in the initial polymerase chain reaction (PCR) cycles as well as enabling removal of PCR duplicates and allows variant calling at much lower thresholds than non–error-corrected NGS.[1,3,4]

Details on the NGS/MRD analysis software/algorithms used and their settings

Raw binary base call files from NovaSeq 6000® were demultiplexed on a per-sample level with BCL Convert (version 2.4.0; Illumina, San Diego, CA) using 5 bp for UMIs. Subsequently, resulting FASTQ files were loaded into DRAGEN Enrichment (version 4.2.4; Illumina, San Diego, CA).

We used the manufacturer's default parameters with a custom target bed file with the following settings

- Variant caller mode: somatic
- Somatic variant frequency call threshold (percentage): 0.1
- Somatic variant frequency filter threshold (percentage): 0.1
- UMI library type: NONRANDOM-DUPLEX
- UMI-aware variant calling: high depth
- UMI min supporting reads: 15
- UMI nonrandom whitelist
    - AACAC
    - AAGGA
    - AATGC
    - ACAAC

- ACTAG
- AGCAT
- AGTAC
- ATACG
- ATCTC
- CACTG
- CAGAC
- CAGTA
- CATGA
- CGAAT
- CGATA
- CGGTT
- CGTGT
- CTTGG
- GCATA
- GCCAT
- GCTAA
- GCTGT
- GTCAC
- GTCGT
- GTGAG
- GTGTC
- TACGA
- TCCTA
- TCGTG
- TGTCG

- TGTGC
- TTGGC

- Combine phased variants: 8

References

1. Patkar N, Kakirde C, Shaikh AF *et al*. Clinical impact of panel-based error-corrected next generation sequencing versus flow cytometry to detect measurable residual disease (MRD) in acute myeloid leukemia (AML). *Leukemia.* 2021;35(5):1392-1404.
2. Vonk CM, Al Hinai ASA, Hanekamp D *et al.* Molecular minimal residual disease detection in acute myeloid leukemia. *Cancers (Basel).* 2021;13(21):5431.
3. Hasserjian RP, Steensma DP, Graubert TA *et al*. Clonal hematopoiesis and measurable residual disease assessment in acute myeloid leukemia. *Blood*. 2020;135(20):1729-1738.
4. Thol F, Gabdoulline R, Liebich A *et al*. Measurable residual disease monitoring by NGS before allogeneic hematopoietic cell transplantation in AML. *Blood*. 2018;132(16):1703-1713.

Supplemental results for the MRD analyses

Non-MRD panel baseline mutations in patients with an MRD-negative response

One patient had mutations in *BCOR, CSF3R, PHF6,* and isocitrate dehydrogenase 1 (*IDH1*); 1 patient had mutations in *BCOR, BCORL1*, and *IDH1;* and 1 patient had mutations in *BCOR* and *IDH1*. For all 3 patients, only the mutations in *IDH1* were able to be followed during treatment. One patient had mutations in *FLT3, IDH1, RUNX1, SRSF2,* and *SMC3*, and all mutations could be followed aside from the mutation in *SMC3.*

Supplemental Table 1. 51-gene AML diagnostic panel (gene/region of interest)

ASXL1/ E12, E13	*ASXL2*/ E12, E13	*BCOR**	*BCORL1**	*CALR*/ E09	*CBL**	*CEBPA**
CSF3R/ E14–E17	*CSNK1A1*/ E03, E04	*CUX1**	*DDX41**	*DNMT3A**	*ETNK1*/ E03	*ETV6**
*EZH2**	*FLT3*/ E14–E20	*GATA1**	*GATA2**	*IDH1*/ E04, E07	*IDH2*/ E04, E07	*JAK2**
*KIT**	*KRAS**	*MPL**	*NF1**	*NPM1*/ E11	*NRAS**	*PDGFRA**
*PDGFRB**	*PHF6**	*PIGA**	*PPM1D**	*PTPN11**	*RAD21**	*RUNX1**
SETBP1/ E04	*SF1**	*SF3A1**	*SF3B1*/ E13–E16	*SH2B3**	*SMC1A**	*SMC3**
SRSF2/ E01	*STAG2**	*TET2**	*TP53**	*UBA1**	*U2AF1*/ E02, E06	*U2AF2*/ E02, E06
WT1/ E07, E09	*ZRSR2**					

*Complete coding sequence analyzed.
AML, acute myeloid leukemia.

Supplemental Table 2. Munich Leukemia Laboratory AML MRD 26-gene panel (gene/region of interest)

ASXL1/<br>E13	*CALR*/<br>E09	*CEBPA**	*DDX41**	*DNMT3A**
*ETV6**	*EZH2**	*FLT3*/<br>E14–E16, E20	*IDH1*/<br>E04	*IDH2*/<br>E04
JAK2/<br>E12, E14	*KIT*/<br>E08, E11, E17	*KRAS*/<br>E02, E03	*MPL*/<br>E10	*NPM1*/<br>E11
NRAS/<br>E02, E03	*PTPN11*/<br>E03, E13	*RAD21**	*RUNX1**	*SF3B1*/<br>E13–E16
SRSF2/<br>E01	*STAG2**	*TET2**	*TP53*/<br>E02–E10	*U2AF1*/<br>E02, E06
WT1/<br>E07, E09				

*Complete coding sequence analyzed.
AML, acute myeloid leukemia; MRD, measurable residual disease.

Supplemental Table 3. Baseline demographic and clinical characteristics in the ITT population

**Characteristic**	**Ivosidenib + azacitidine N=73**	**Placebo + azacitidine N=75**
Median age (range), years	76.0 (58.0–84.0)	76 (45.0–94.0)
Sex, n (%)		
Male	42 (58)	38 (51)
Female	31 (42)	37 (49)
Race or ethnic group, n (%)		
Asian	15 (21)	19 (25)
White	12 (16)	12 (16)
Black	0	2 (3)
Other/not reported	46 (63)	42 (56)
ECOG performance status, n (%)		
0	14 (19)	10 (13)
1	32 (44)	41 (55)
2	27 (37)	24 (32)
Disease history per investigator, n (%)		
Primary AML	55 (75)	53 (71)
Secondary AML	18 (25)	22 (29)
History of myeloproliferative neoplasms	4 (5)	8 (11)
World Health Organization classification, n (%)		
AML with recurrent genetic abnormalities	16 (22)	24 (32)
AML with myelodysplasia-related changes	28 (38)	27 (36)
Therapy-related myeloid neoplasms	1 (1)	1 (1)
Not otherwise specified	28 (38)	23 (31)

IDH1 mutation type, n (%)		
R132C	46 (63)	52 (69)
R132G	6 (8)	4 (5)
R132H	14 (19)	12 (16)
R132L	3 (4)	0
R132S	2 (3)	6 (8)
Wildtype/missing	2 (3)	1 (1)
Cytogenetic risk status, n (%)		
Favorable	3 (4)	7 (9)
Intermediate	49 (67)	45 (60)
Poor	16 (22)	20 (27)
Other/missing	5 (7)	3 (4)
Median bone marrow blast level, % (range)	53.5 (32.5–75.0)	48.5 (33.0–72.0)

AML, acute myeloid leukemia; ECOG, Eastern Cooperative Oncology Group; *IDH1*, isocitrate dehydrogenase 1; ITT, intention-to-treat.

**Supplemental Table 4. Subsequent anticancer therapies in the ITT population**		
	**Ivosidenib + azacitidine N=73**	**Placebo + azacitidine N=75**
Any subsequent anticancer therapy, n (%)	19 (26)	23 (31)
Type of therapy, n (%)*		
BCL2 inhibitor	5 (7)	8 (11)
Chemotherapy	8 (11)	15 (20)
HSCT conditioning	5 (7)	2 (3)
Hypomethylating agent	4 (6)	5 (7)
IDH1 inhibitor	1 (1)	4 (5)
Immunotherapy	2 (3)	0
Other	3 (4)	2 (3)
Other targeted therapy	5 (7)	0

*The most commonly reported therapies with antimetabolites were azacitidine (n=17, 11.5%), cytarabine (n=11, 7.4%), fludarabine (n=8, 5.4%), and decitabine (n=5, 3.4%). A total of 17 patients (11.5%) received venetoclax-based regimens as a subsequent anticancer therapy, 6 (8.2%) from the ivosidenib-azacitidine arm and 11 (14.7%) from the placebo-azacitidine arm. BCL2, B-cell lymphoma 2; HSCT, hematopoietic stem cell transplantation; *IDH1*, isocitrate dehydrogenase 1; ITT, intention-to-treat.

Supplemental Table 5. Baseline demographics and disease characteristics in ivosidenib-azacitidine–treated patients in the MRD-evaluable population, overall and according to MRD response at the 0.1% threshold

	MRD evaluable N=33	MRD_{neg} response N=10	MRD_{pos} response N=23
Median age, years (range)	76.0 (58–84)	77.0 (65–84)	76.0 (58–82)
Male/female, n (%)	18 (54.5)/ 15 (45.5)	3 (30.0)/ 7 (70.0)	15 (65.2)/ 8 (34.8)
Disease type, n (%)			
De novo AML	24 (72.7)	8 (80.0)	16 (69.6)
Secondary AML	9 (27.3)	2 (20.0)	7 (30.4)
History of MDS	7 (21.2)	2 (20.0)	5 (21.7)
History of MPN	1 (3.0)	0	1 (4.3)
Other	1 (3.0)	0	1 (4.3)
ECOG PS, n (%)			
0	5 (15.2)	1 (10.0)	4 (17.4)
1	15 (45.5)	5 (50.0)	10 (43.5)
2	13 (39.4)	4 (40.0)	9 (39.1)
IDH1 R132 variant, n patients (%)			
R132C	26 (78.8)	7 (70.0)	19 (82.6)
R132G	3 (9.1)	1 (10.0)	2 (8.7)
R132H	3 (9.1)	1 (10.0)	2 (8.7)
R132L	1 (3.0)	1 (10.0)	0
ELN 2022 risk			
Favorable	2 (6.1)	1 (10.0)	1 (4.3)
Intermediate	6 (18.2)	3 (30.0)	3 (13.0)
Adverse	19 (57.6)	5 (50.0)	14 (60.9)
Unknown	6 (18.2)	1 (10.0)	5 (21.7)

AML, acute myeloid leukemia; ECOG PS, Eastern Cooperative Oncology Group performance status; ELN, European LeukemiaNet; *IDH1*, isocitrate dehydrogenase 1; MDS, myelodysplastic syndromes; MPN, myeloproliferative neoplasms; MRD, measurable residual disease; MRD_{neg}, MRD negative; MRD_{pos}, MRD positive.

Supplemental Table 6. Presence or absence of baseline mutations and correlation with remaining MRD_{pos} or converting to MRD negativity*

Gene	Number of patients with a baseline alteration		Number of patients without a baseline alteration		*p* value	Odds ratio
	Remaining MRD_{pos}	Converting to MRD negativity	Remaining MRD_{pos}	Converting to MRD negativity		
ASXL1	8	1	10	8	.192501	6.017758
CEBPA	2	1	16	8	1	1
DNMT3A	5	6	13	3	.096873	0.206105
EZH2	2	1	16	8	1	1
FLT3	3	1	15	8	1	1.574063
IDH1	18	9	0	0	1	0
KRAS	1	0	17	9	1	Inf
NPM1	2	1	16	8	1	1
NRAS	1	2	17	7	.250256	0.219973
RAD21	0	1	18	8	.333333	0
RUNX1	6	1	12	8	.363211	3.824424
SRSF2	7	1	11	8	.201119	4.827996
STAG2	2	2	16	7	.581538	0.452336
TET2	3	0	15	9	.529231	Inf
TP53	1	0	17	9	1	Inf
U2AF1	4	0	14	9	.267692	Inf
WT1	1	0	17	9	1	Inf

*According to Fisher's exact test.
Inf, infinite; MRD, measurable residual disease; MRD_{pos}, MRD positive.

**Supplemental Table 7. Baseline demographics and disease characteristics in ivosidenib-azacitidine–treated MRD-evaluable patients according to MRD response at a 1% VAF threshold**		
	**$MRD_{<1\%}$ response N=20**	**$MRD_{\geq1\%}$ response N=13**
Median age, years (min–max)	74.5 (65–84)	77.0 (58–82)
Male/female, n (%)	9 (45.0)/11 (55.0)	9 (69.2)/4 (30.8)
Disease type, n (%)		
De novo AML	17 (85.0)	7 (53.8)
Secondary AML	3 (15.0)	6 (46.2)
History of MDS	3 (15.0)	4 (30.8)
History of MPN	0	1 (7.7)
Other	0	1 (7.7)
ECOG PS, n (%)		
0	3 (15.0)	2 (15.4)
1	9 (45.0)	6 (46.2)
2	8 (40.0)	5 (38.5)
IDH1 R132 variant, n patients (%)		
R132C	17 (85.0)	9 (69.2)
R132G	1 (5.0)	2 (15.4)
R132H	1 (5.0)	2 (15.4)
R132L	1 (5.0)	0
ELN 2022 risk		
Favorable	1 (5.0)	1 (7.7)
Intermediate	4 (20.0)	2 (15.4)
Adverse	13 (65.0)	6 (46.2)
Unknown	2 (10.0)	4 (30.8)

AML, acute myeloid leukemia; ECOG PS, Eastern Cooperative Oncology Group performance status; ELN, European LeukemiaNet; *IDH1*, isocitrate dehydrogenase 1; MDS, myelodysplastic syndromes; MPN, myeloproliferative neoplasms; MRD, measurable residual disease; $MRD_{<1\%}$, baseline mutations <1% MRD VAF; $MRD_{\geq1\%}$, baseline mutations ≥1% MRD VAF; VAF, variant allele frequency.

Supplemental Figure 1. Variant allele frequency distribution of *IDH1* mutations in bone marrow in the ITT population. *IDH1*, isocitrate dehydrogenase 1; ITT, intention-to-treat.

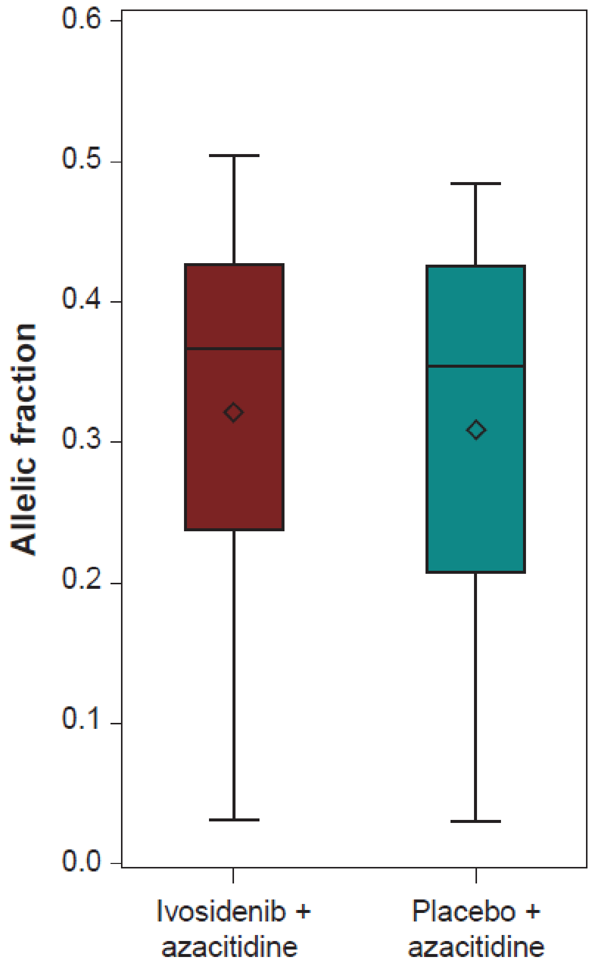

Supplemental Figure 2. Disposition of patients in the MRD analyses.
*CRi included CRp. †MRD-evaluable was defined as having at least 1 on-treatment bone marrow mononuclear cell sample available with a corresponding response assessment. CRi, complete remission with incomplete hematologic recover; CRp, complete remission with incomplete platelet recovery; MRD, measurable residual disease; MRD_{neg}, MRD negative; MRD_{pos}, MRD positive.

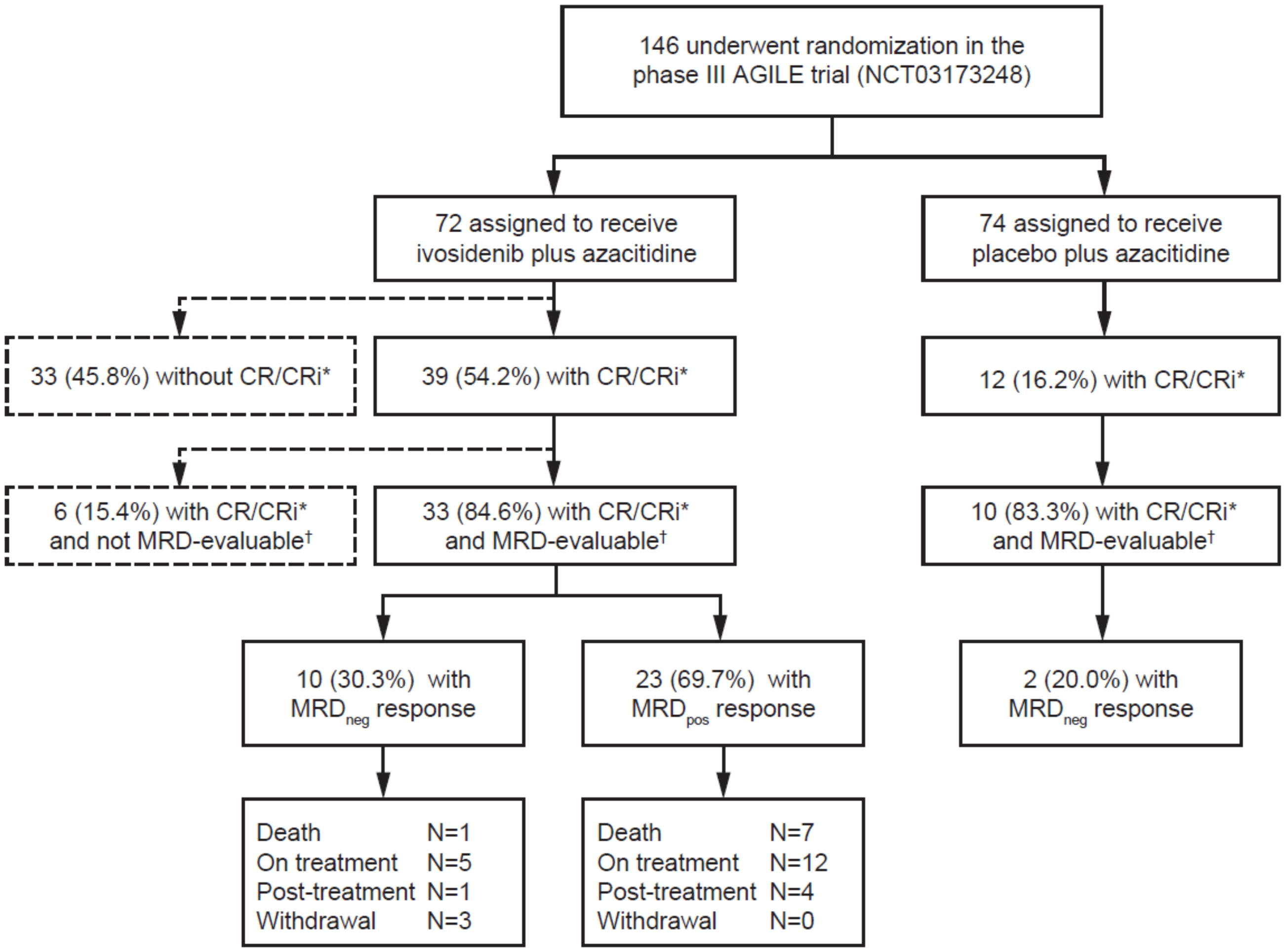

Supplemental Figure 3. Overall survival in patients treated with ivosidenib-azacitidine and placebo-azacitidine according to baseline *IDH1* VAF. CI, confidence interval; *IDH1*, isocitrate dehydrogenase 1; NE, not evaluable; OS, overall survival; VAF, variant allele frequency.

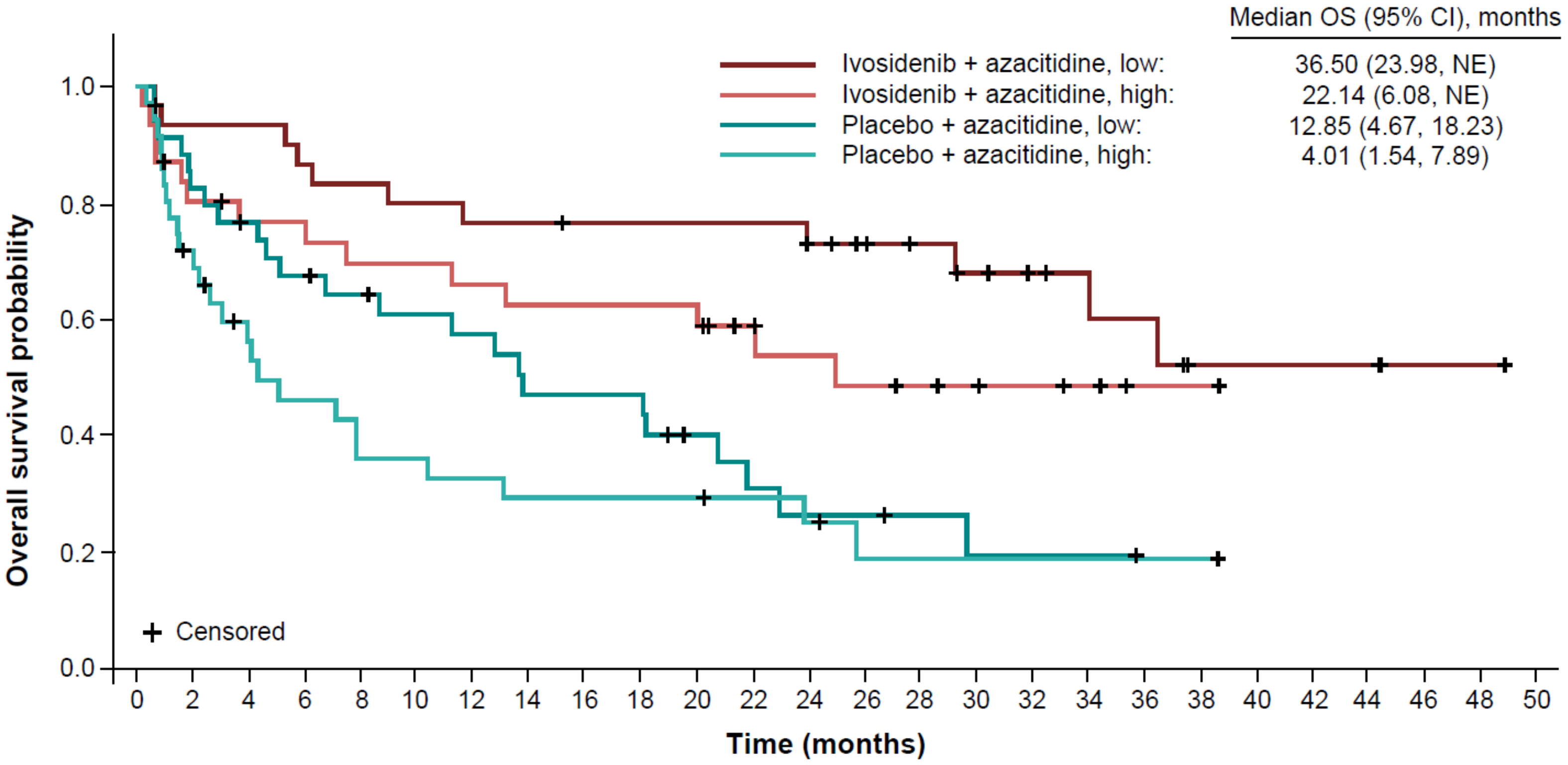

No. patients at risk:

Ivosidenib + azacitidine, low:	27	24	24	22	21	20	19	19	18	18	18	18	16	14	12	10	8	7	6	3	3	3	3	1	1	0
Ivosidenib + azacitidine, high:	27	20	18	18	16	16	15	14	14	14	14	10	8	7	6	5	4	3	1	1	0					
Placebo + azacitidine, low:	30	24	21	18	16	14	13	10	10	10	6	4	3	3	2	1	1	1	0							
Placebo + azacitidine, high:	31	20	14	10	7	7	6	5	5	5	5	4	3	1	1	1	1	1	1	1	0					

Supplemental Figure 4. Hematology outcomes for patients treated with ivosidenib-azacitidine or placebo-azacitidine. Median absolute hemoglobin levels (A), platelet counts (B), and neutrophil counts (C) over time. C, cycle; D, day.

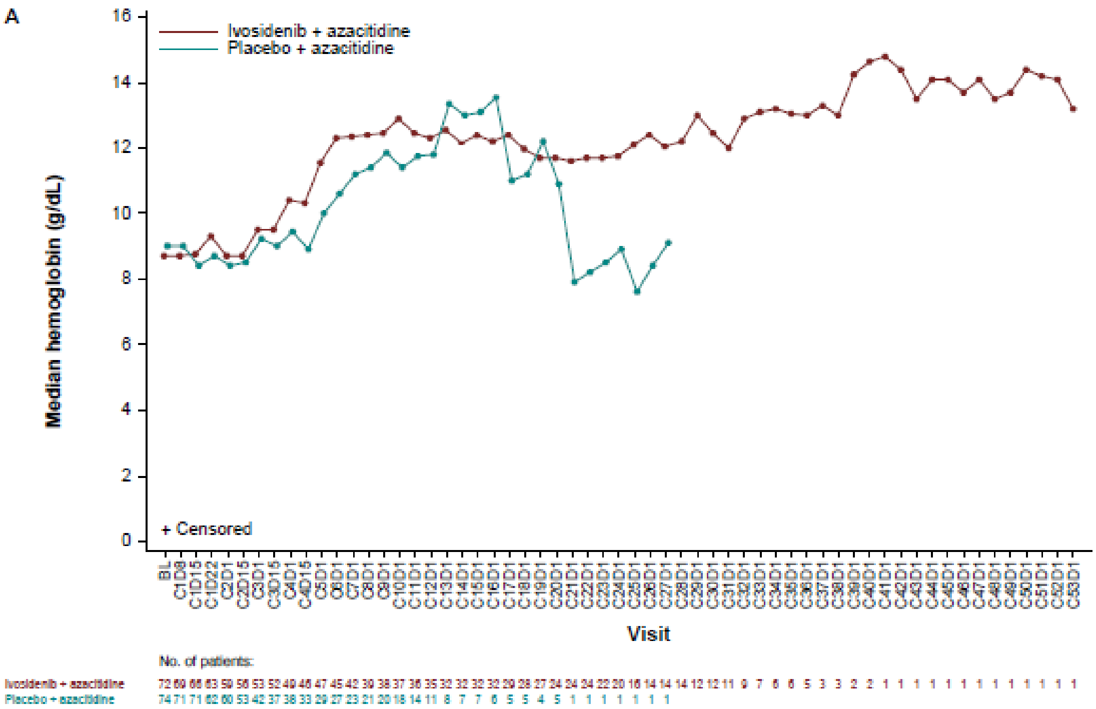

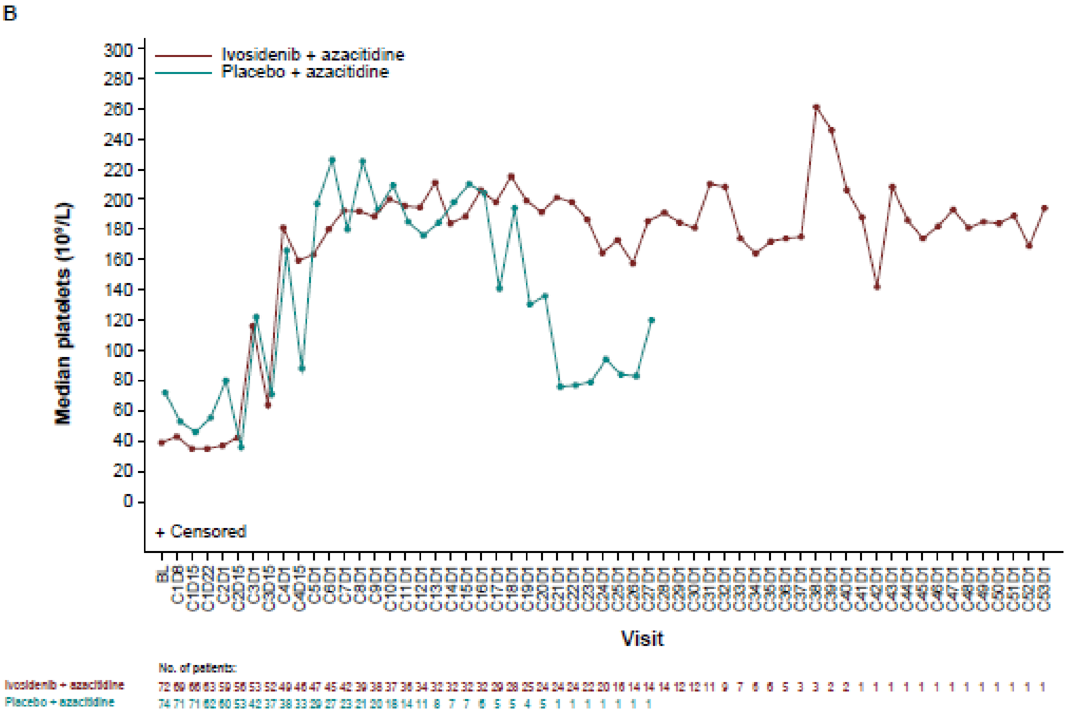

C

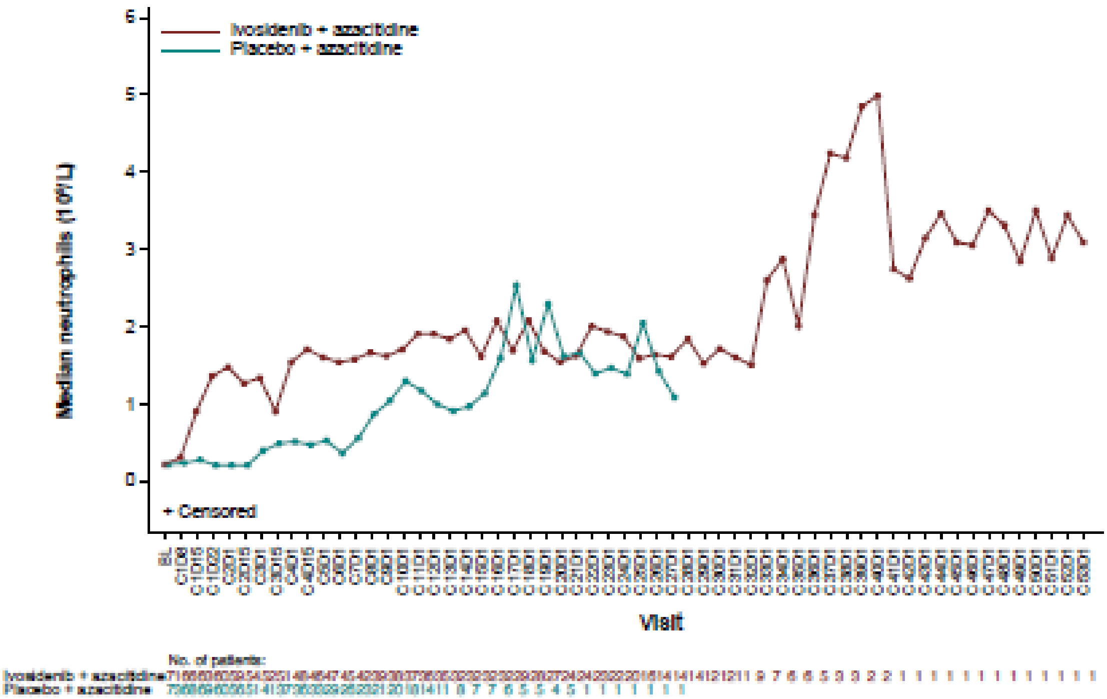
Ivosidenib + azacitidine
Placebo + azacitidine
Median neutrophils (10⁹/L)
+ Censored
Visit
No. of patients:
Ivosidenib + azacitidine
Placebo + azacitidine

Supplemental Figure 5. Time to MRD_{neg} response in ivosidenib-azacitidine–treated patients (N=10). C, cycle; D, day; MRD, measurable residual disease; MRD_{neg}, MRD negative.

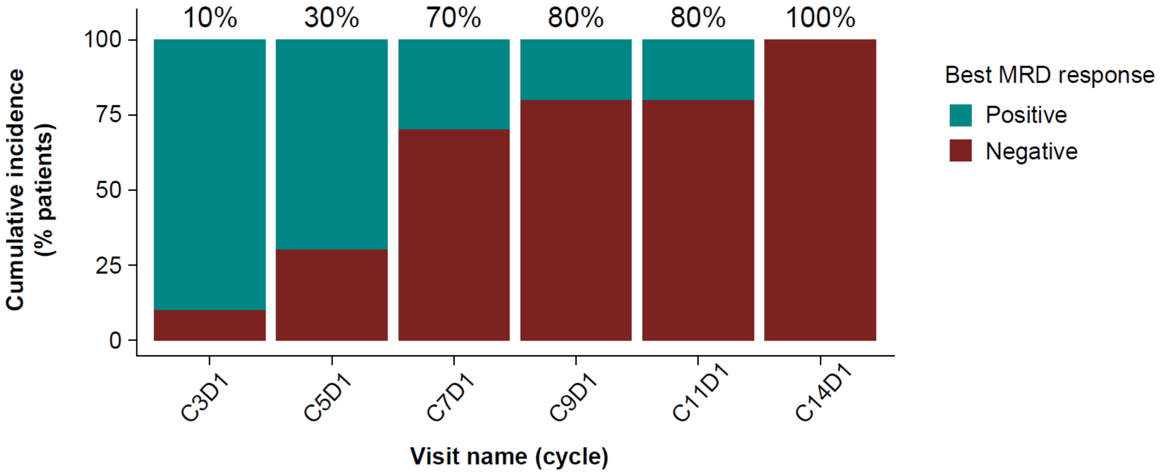

Supplemental Figure 6. Best MRD response in ivosidenib-azacitidine–treated MRD-evaluable patients (N=33) according to baseline mutation status. Response according to baseline m*IDH1* VAF (A), to inference of m*IDH1* clonality (B), and to the number of variants (C). m*IDH1*, mutant isocitrate dehydrogenase 1; MRD, measurable residual disease; VAF, variant allele frequency.

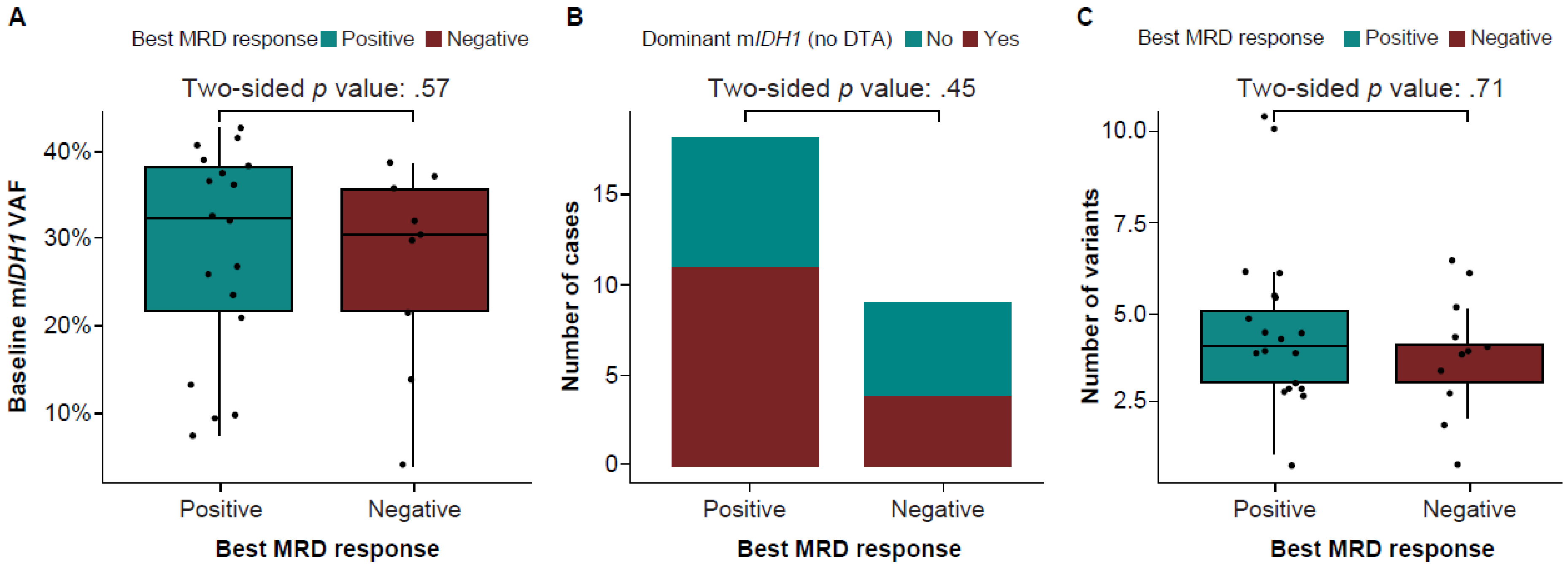

Supplemental Figure 7. Longitudinal evaluation of genes mutated at baseline and present in at least 1 patient in ivosidenib-azacitidine–treated MRD-evaluable patients (N=33).

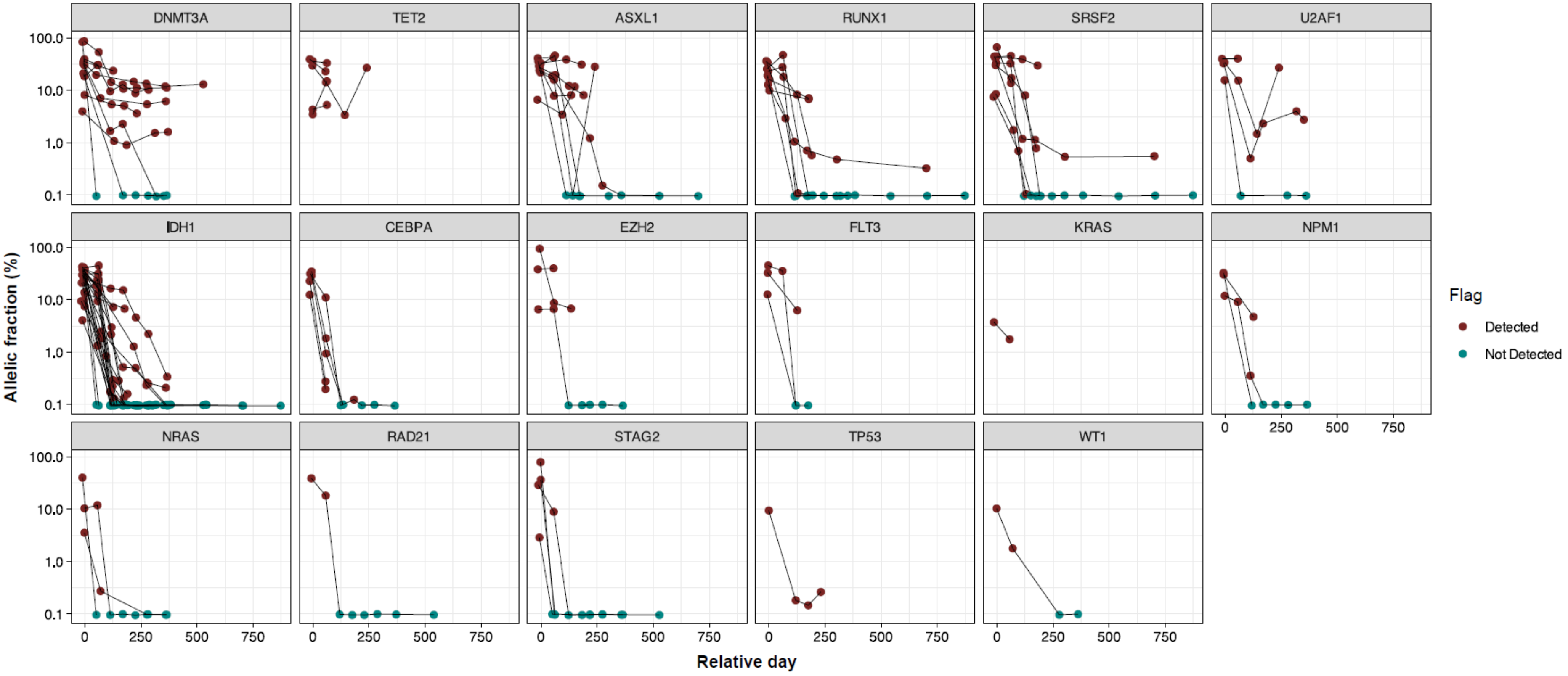

Supplemental Figure 8. Swimlane plot of MRD status over time in ivosidenib-azacitidine–treated MRD-evaluable patients (N=33).
*One patient converted from an MRD_{neg} to an MRD_{pos} response ("MRD relapse") 169 days prior to overt clinical relapse; the other 4 patients were MRD_{pos} at all prior assessments. †CRi included CRp. CR, complete remission; CRi, complete remission with incomplete hematologic recovery; CRp, complete remission with incomplete platelet recovery; MRD, measurable residual disease; MRD_{neg}, MRD negative; MRD_{pos}, MRD positive; NE, not evaluable.

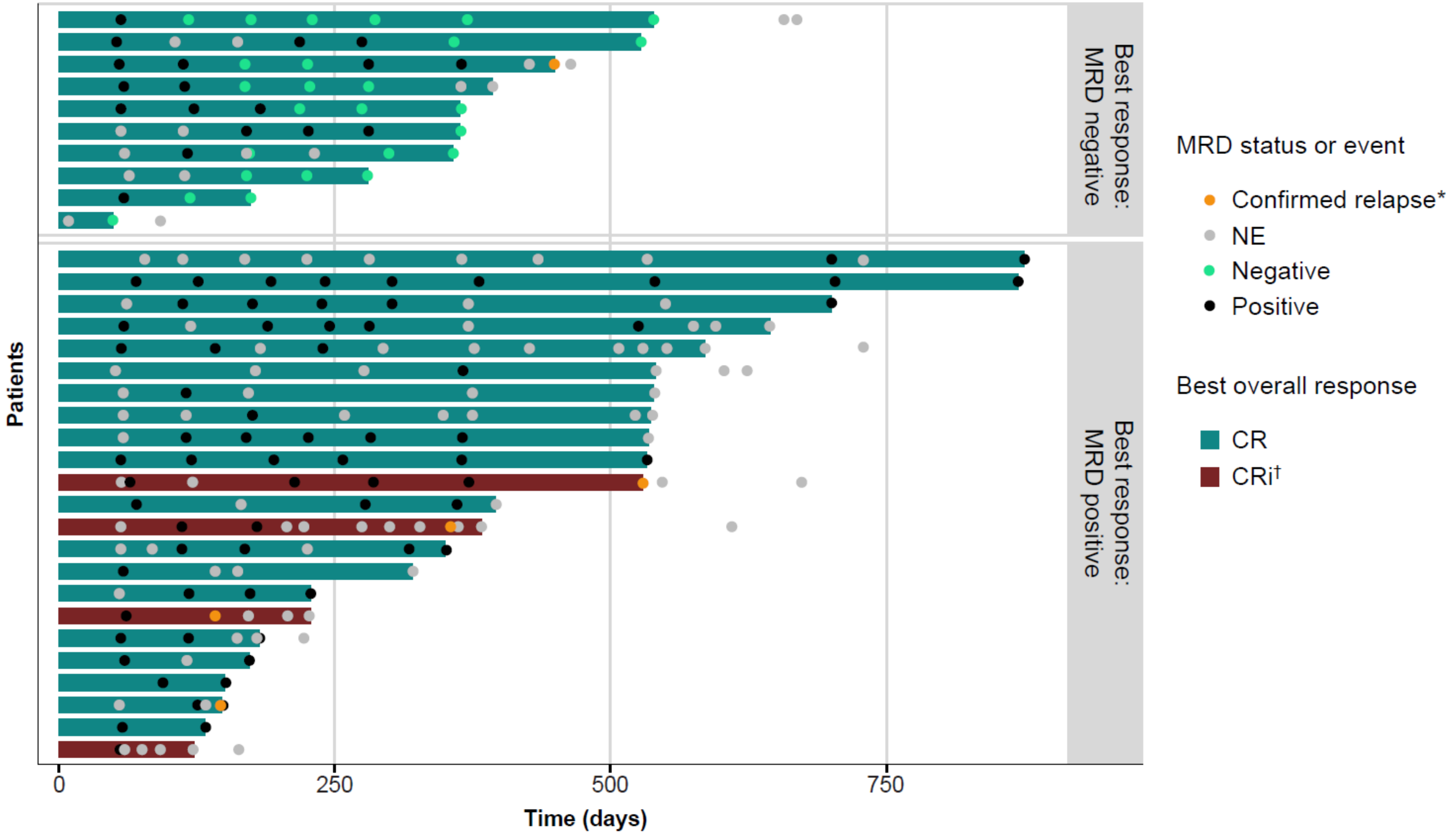

Supplemental Figure 9. Duration of clinical outcomes in ivosidenib-azacitidine–treated MRD-evaluable patients according to MRD status (N=33). (A) Duration of CR + CR$_i$ according to MRD response status. (B) Duration of CR + CR$_i$ in patients with MRD$_{<1\%}$ vs patients with MRD$_{\geq 1\%}$. (C) EFS according to MRD response status. (D) EFS in patients with MRD$_{<1\%}$ vs patients with MRD$_{\geq 1\%}$. *CRi included CRp. CI, confidence interval; CR, complete remission; CRi, complete remission with incomplete hematologic recovery; CRp, complete remission with incomplete platelet recovery; EFS, event-free survival; MRD, measurable residual disease; MRD$_{<1\%}$, baseline mutations <1% MRD VAF; MRD$_{\geq 1\%}$, baseline mutations ≥1% MRD VAF; MRD$_{neg}$, MRD negative; MRD$_{pos}$, MRD positive; NE, not evaluable; VAF, variant allele frequency.

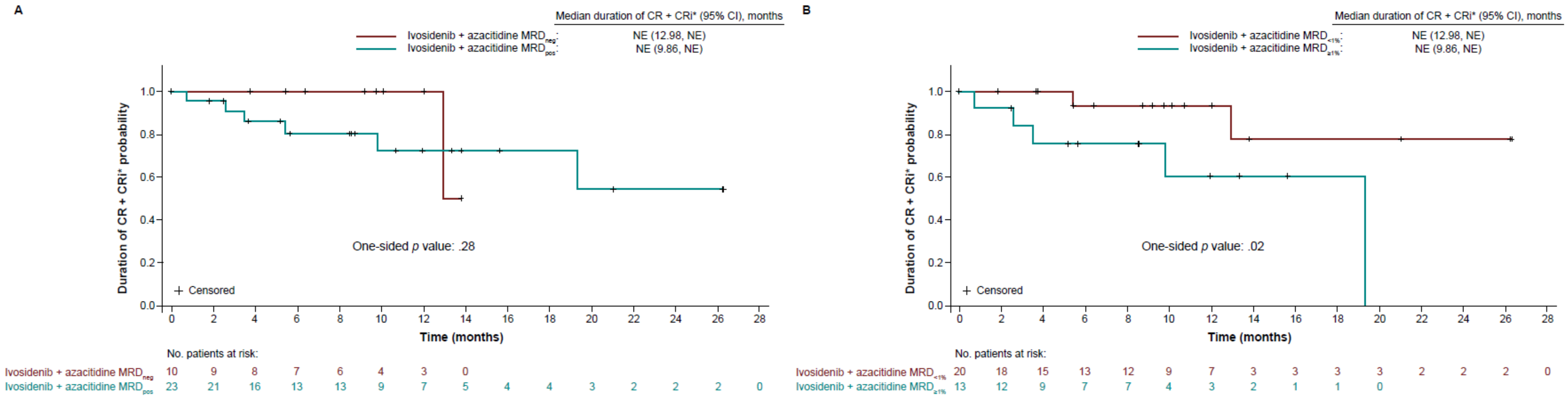

C

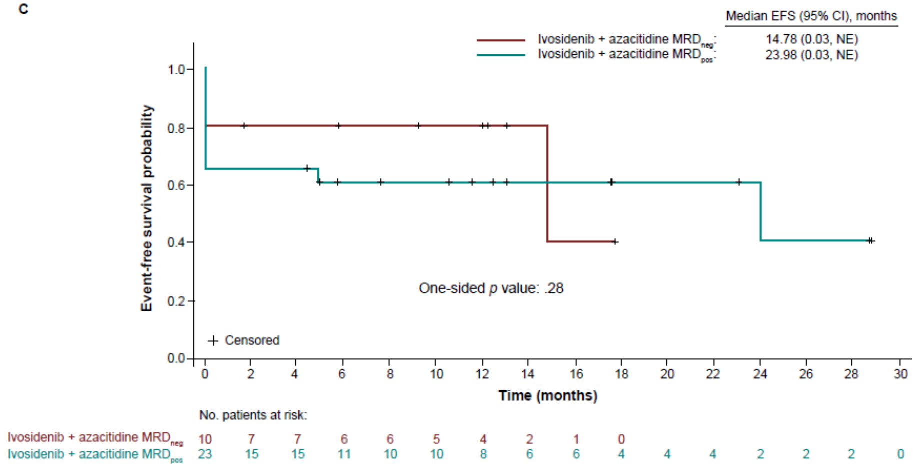
Median EFS (95% CI), months
Ivosidenib + azacitidine MRD$_{neg}$: 14.78 (0.03, NE)
Ivosidenib + azacitidine MRD$_{pos}$: 23.98 (0.03, NE)
Event-free survival probability
1.0
0.8
0.6
0.4
0.2
0.0
One-sided *p* value: .28
+ Censored
0 2 4 6 8 10 12 14 16 18 20 22 24 26 28 30
Time (months)
No. patients at risk:
Ivosidenib + azacitidine MRD$_{neg}$ 10 7 7 6 6 5 4 2 1 0
Ivosidenib + azacitidine MRD$_{pos}$ 23 15 15 11 10 10 8 6 6 4 4 4 2 2 2 0

D

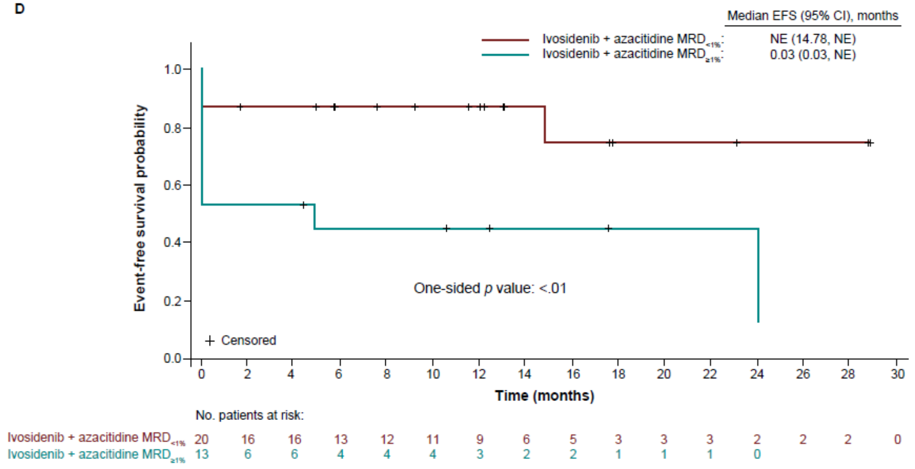
Median EFS (95% CI), months
Ivosidenib + azacitidine MRD$_{<1\%}$: NE (14.78, NE)
Ivosidenib + azacitidine MRD$_{\geq 1\%}$: 0.03 (0.03, NE)
Event-free survival probability
1.0
0.8
0.6
0.4
0.2
0.0
One-sided *p* value: <.01
+ Censored
0 2 4 6 8 10 12 14 16 18 20 22 24 26 28 30
Time (months)
No. patients at risk:
Ivosidenib + azacitidine MRD$_{<1\%}$ 20 16 16 13 12 11 9 6 5 3 3 3 2 2 2 0
Ivosidenib + azacitidine MRD$_{\geq 1\%}$ 13 6 6 4 4 4 3 2 2 1 1 1 0

Supplemental Figure 10. Exploratory analysis for an alternative definition of EFS according to MRD response in ivosidenib-azacitidine–treated patients (N=33).

Alternative EFS was defined as the time from randomization until disease progression, relapse following CR or CRi or death from any cause, or treatment failure, with the definition of treatment failure being amended to include failure of ivosidenib plus azacitidine to induce CR, CRi, or morphologic leukemia-free state within 24 weeks of treatment.

(A) Alternative EFS by MRD response. (B) Alternative EFS in patients with MRD$_{<1\%}$ vs patients with MRD$_{\geq 1\%}$. CI, confidence interval; CR, complete remission; CRi, complete remission with incomplete hematologic recovery; EFS, event-free survival; MRD, measurable residual disease; MRD$_{<1\%}$, baseline mutations <1% MRD VAF; MRD$_{\geq 1\%}$, baseline mutations ≥1% MRD VAF; MRD$_{neg}$, MRD negative; MRD$_{pos}$, MRD positive; NE, not evaluable; VAF, variant allele frequency MRD$_{neg}$, MRD negative; MRD$_{pos}$, MRD positive; NE, not evaluable; VAF, variant allele frequency.

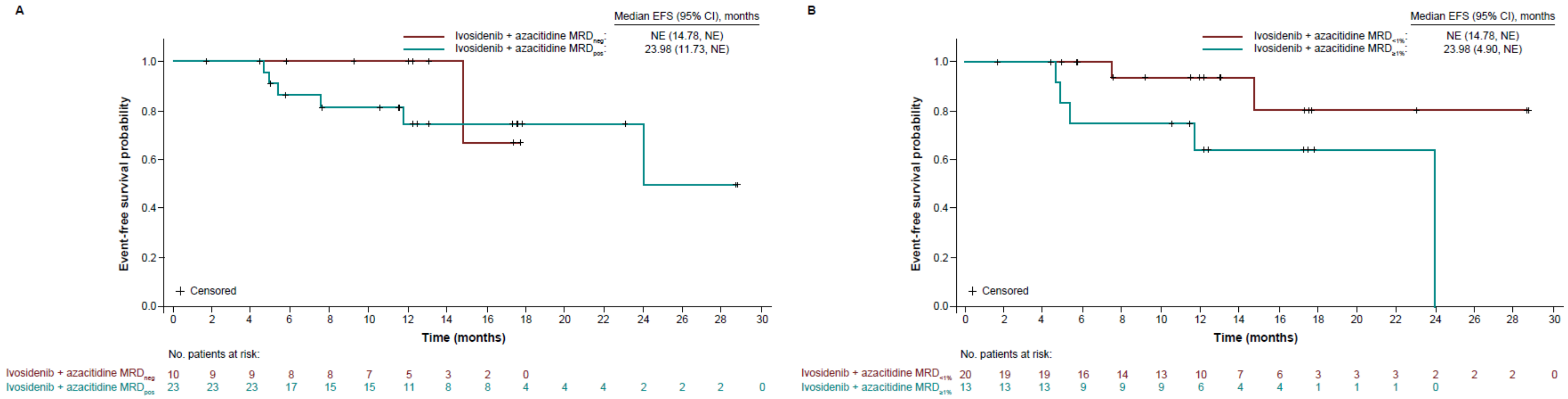

Supplement: Supplemental Methods, References, Tables, and Figures [file BLOODA_ADV-2025-016399-mmc1.pdf]
